# Machine learning for myocarditis diagnosis using cardiovascular magnetic resonance: a systematic review, diagnostic test accuracy meta-analysis, and comparison with human physicians

**DOI:** 10.1007/s10554-025-03497-5

**Published:** 2025-09-09

**Authors:** Paweł Łajczak, Oguz Kagan Sahin, Jakub Matyja, Luis Rene Puglla Sanchez, Iqbal Farhan Sayudo, Ayesha Ayesha, Vitor Lopes, Mir Wajid Majeed, Mrinal Murali Krishna, Meghna Joseph, Mable Pereira, Ogechukwu Obi, Railla Silva, Caterina Lecchi, Michele Schincariol

**Affiliations:** 1 Medical University of Silesia, Katowice, Poland; 2 Edremit State Hospital, Balikesir, Turkey; 3 TU Delft, Delft, Netherlands; 4 Clinico Lozano Blesa University Hospital, Zaragoza, Spain; 5 Medical Research Unit, Universitas Syiah Kuala, Banda Aceh, Indonesia; 6 Shifa College of Medicine, Islamabad, Pakistan; 7 Federal University of Amazonas, Manaus, Brazil; 8 Government Medical College, Srinagar, India; 9 Medical College, Thiruvananthapuram, India; 10 Lincoln American University School of Medicine, Georgetown, Guyana; 11 New York Institute of Technology College of Osteopathic Medicine, Old Westbury, New York, USA; 12 Federal University of Ceara, Fortaleza, Brazil; 13 University of Svizzera Italiana, Lugano, Switzerland; 14 Klinikum Fürth, Friedrich-Alexander-University Erlangen- Nürnberg, Fürth, Germany

**Keywords:** Machine learning, Diagnostic accuracy, Myocarditis, CMR

## Abstract

Myocarditis is an inflammation of heart tissue. Cardiovascular magnetic resonance imaging (CMR) has emerged as an important non-invasive imaging tool for diagnosing myocarditis, however, interpretation remains a challenge for novice physicians. Advancements in machine learning (ML) models have further improved diagnostic accuracy, demonstrating good performance. Our study aims to assess the diagnostic accuracy of ML in identifying myocarditis using CMR. A systematic search was performed using PubMed, Embase, Web of Science, Cochrane, and Scopus to identify studies reporting the diagnostic accuracy of ML in the detection of myocarditis using CMR. The included studies evaluated both image-based and report-based assessments using various ML models. Diagnostic accuracy was estimated using a Random-Effects model (R software). We found a total of 141 ML model results from a total of 12 studies, which were included in the systematic review. The best models achieved 0.93 (95% Confidence Interval (CI) 0.88–0.96) sensitivity and 0.95 (95% CI 0.89–0.97) specificity. Pooled area under the curve was 0.97 (95% CI 0.93–0.98). Comparisons with human physicians showed comparable results for diagnostic accuracy of myocarditis. Quality assessment concerns and heterogeneity were present. CMR augmented using ML models with advanced algorithms can provide high diagnostic accuracy for myocarditis, even surpassing novice CMR radiologists. However, high heterogeneity, quality assessment concerns, and lack of information on cost-effectiveness may limit the clinical implementation of ML. Future investigations should explore cost-effectiveness and minimize biases in their methodologies.

## Introduction

Myocarditis is a cardiovascular condition, characterized by inflammation of cardiomyocytes as a result of the immune response and histopathological formation of lymphocytic infiltrates with myocardial necrosis [1]. Kong et al. reported an increased global burden of myocarditis, with an incidence of 1,268,000 cases and 32,450 deaths in 2019 [2].

Myocarditis has a wide range of clinical presentations, from an entirely asymptomatic state to cardiogenic shock and cardiac arrest, making early diagnosis a fundamental step in improving the short- and long-term prognosis for these patients. Endomyocardial biopsy (EMB) is an invasive diagnostic method, so other tools have been developed as first-line tests for early diagnosis, notably cardiac magnetic resonance imaging (CMR) [3].

CMR has emerged as a powerful non-invasive imaging modality for the evaluation of myocarditis [4–6]. Its versatility in assessing myocardial function and tissue characterization, particularly in identifying myocardial fibrosis, and scarring, makes it a preferred tool for proper diagnosis, investigating novel treatment strategies and monitoring disease progression [4, 6]. More specifically, CMR may provide information for myocarditis diagnosis, for example the presence of new regional wall motion, abnormalities of systolic or diastolic functions, pericardial effusion, increased wall thickness, hyperaemia, or oedema [7].

In daily practice, CMR images are interpreted manually and are prone to inter-observer subjective interpretation. In recent years, machine learning (ML) methods, such as neural networks (NNs), deep learning (DL) algorithms, or large language models (LLMs), have been developed to support healthcare professionals in their decision-making process. ML aids by reducing subjectivity, enhancing diagnostic speed, and increasing consistency of results, especially in myocarditis [8–10].

ML techniques are divided into supervised, unsupervised, semi-supervised, and reinforcement learning techniques [8]. Traditional ML methods rely on finding optimal boundaries between classes, like in support vector machines (SVMs) [10]. Notably, NNs, a subset of ML techniques, are emerging, due to their ability to solve complex tasks in medicine [10]. For example, convolutional NNs (CNNs), are a group of high-performance DL models capable of processing massive sets of data, including CMR images, with the advantage of automatically learning spatial features, from low-to high-level patterns [11].

Different ML techniques in CMR diagnosis have been proposed for myocarditis diagnosis; however, the diagnostic performance remains an area that requires deeper analysis of accuracy [10]. This systematic review and diagnostic test accuracy (DTA) meta-analysis aims to synthesize the available evidence on the diagnostic accuracy of ML diagnosis for CMR assessment of myocarditis.

## Methods

### Adherence to guidelines

To conduct this DTA meta-analysis, this paper followed the systematic review guidelines for conducting diagnostic synthesis: Cochrane Handbook for Systematic Reviews of Interventions and the Preferred Reporting Items for Systematic reviews and Meta-Analyses (PRISMA) statement with DTA extension for reporting DTA systematic review papers [12, 13]. To ensure transparency, the protocol for this study was prospectively registered in the PROSPERO database (unique PROSPERO ID number: CRD42024593076).

### Inclusion and exclusion criteria for the studies

The main goal of this study was to assess the diagnostic performance of ML models, therefore for this meta-analysis studies were included if they meet the following criteria: (1) studies (experimental, cohort, randomized trials, or observational) exploring the diagnostic accuracy and evaluating ML model performance in CMR (imaging or reports) for myocarditis diagnosis; (2) included patients (experimental data) diagnosed with or suspected of myocarditis; (3) applied at least one of the ML family techniques (ML, DL, NNs, CNNs, LLMs, etc.) for myocarditis assessment via CMR (either reports or imaging); (4) reported at least one diagnostic accuracy metric, including sensitivity, specificity, F-score, precision, accuracy, or area under the curve (AUC) specifically for myocarditis conditions.

Exclusion criteria were: (1) studies focused on other cardiovascular diseases without myocarditis; (2) absence of relevant diagnostic performance outcomes specifically for myocarditis; and (3) animal studies, editorials, letters to the editor, review papers, case reports, case series, or conference abstracts without full text available.

### Search strategy for database search

To include studies for this systematic review, a comprehensive search strategy was prepared before screening. Five literature databases were searched for this meta-analysis: PubMed, Web of Science, Embase, Scopus, and the Cochrane Library. Records were covered from the default database inception until September 21, 2024.

The search string included keywords, topics, and MeSH terms, which included: ‘deep learning,’ ‘convolutional network,’ ‘computer-aided learning,’ ‘machine learning,’ ‘artificial intelligence,’ ‘myocarditis,’ and ‘inflammatory cardiomyopathy.’

Reference retrieving was performed during full-text screening, and bibliographies of the screened studies were reviewed to find relevant papers. Database records were downloaded and imported into the Zotero screening software (Version 6.0.37). Database files were organized, and potential duplicates were identified and removed with the help of software. The reviewers supervised this process.

### Study screening process and extraction of relevant data

The title and abstract screening process was conducted by two authors independently. The authors were blinded to each other. After the initial screening part was finalized, potential conflicts were resolved with the senior author of this paper. The next step (full-text screening) was performed similarly - two authors screened potentially eligible papers, and conflicts were resolved with the senior author.

After the screening was finalized, the data extraction process from the included papers was conducted by two authors. Disagreements were resolved with the senior author. In case of unclear or missing data, the corresponding authors of the given study were emailed to provide relevant outcome details.

During data extraction, reviewers focused on extracting: (1) country, where the paper was conducted, and study type (design); (2) ML model details including the details of the best performing model and its results, diagnostic accuracy of the other models (if such were provided in the paper), data optimizer technique for ML model, software and hardware used to develop and run ML diagnostic model; (3) details of CMR data including source (institution or online database) of data, image size (only applicable to imaging studies), preprocessing techniques, data augmentation methods (i.e., rotations or contrast), cardiovascular conditions analyzed in the study, number of cases in the dataset and testing sets, number of myocarditis cases, and information who performed reference labelling (gold standard); (4) validation process in the study, including technique of validation (cross-validation, split, or external validation on independent dataset), number of cases in training, validation, and testing sets; (5) diagnostic accuracy metrics including sensitivity, specificity, accuracy, precision, F-score, AUC, and geometric mean score (G-means).

Data extraction was performed in a shared Excel sheet.

### Main outcomes of diagnostic accuracy

This DTA meta-analysis focused on several diagnostic accuracy outcomes. Generally, a value closer to 1 indicates stronger and more accurate diagnostic performance. Sensitivity (recall) is a metric that describes the ability of the model to detect patients with the condition (myocarditis). Specificity, on the other hand, describes the ability to diagnose patients without a condition (non-myocarditis patients). Accuracy is a summary diagnostic accuracy metric, and it is a sum of correct predictions divided by the total sum of all predictions. Precision, known as positive predictive value (PPV), is defined as the number of TP divided by the sum of positive calls (presence of myocarditis). F-score is calculated based on precision and sensitivity, representing both diagnostic parameters in one. AUC value of 0.5 indicates random guessing of myocarditis presence, while a value of 1 suggests strong diagnostic capabilities for myocarditis diagnosis. Finally, G-means is a metric based on sensitivity and specificity.

### Statistical Approach, DTA Meta-Analysis, Univariate and Bivariate Analysis, and Heterogeneity Assessment

Firstly, we summarized the diagnostic accuracy metrics of the ML models from the included studies. Range (lowest and highest), interquartile range, median, mean, and standard deviation values were calculated with the use of R (v. 4.3.3) software.

The diagnostic outcomes were calculated in the DTA meta-analysis. Diagnostic accuracy analysis was performed on confusion matrix values: TP, FN, FP, and TN. Missing confusion matrix values were obtained by either contacting the corresponding authors of the study, or calculating them based on sensitivity, specificity, number of cases in the testing set, and prevalence of myocarditis cases in the testing set. R software (v. 4.3.3) was used to perform all statistical analyses. Meta-analysis (meta) statistical package was used to plot diagnostic accuracy results [14].

DTA outcomes included recall, specificity, AUC, diagnostic odds ratio (DOR), and false positive rate.

Firstly, a univariate model analysis was performed. For sensitivity and specificity, the effect size was calculated, with a heterogeneity metric. Results of univariate analyses were reported, with forest plots for each outcome separately.

Bivariate analysis was reported with AUC. A summary receiver operating characteristic (ROC) curve was used to visualize the included studies and the summary curve. DOR results were presented in form of a forest plot.

In both univariate and bivariate analyses, a random-effects model was used to conduct analyses, as we expected between-study variations due to study design, populations, ML models, CMR protocols, and validation techniques.

Firstly, we ran a meta-analysis with all eligible models. After the overall analysis was performed, a sub-analysis of the best-performing model from each study was done.

Effect size outcomes were reported with ninety-five percent confidence intervals (95% CI). The heterogeneity assessment was done with both the Cochran Q test and the I² value. I² over 50% and p-value < 0.1 for the Cochran Q test suggested presence of variability and evidence for between-study heterogeneity in the analysis.

Finally, the threshold effect influence was analyzed to explore its potential effect on the heterogeneity of the results.

### Risk of bias and quality assessment

The quality assessment (risk of bias) was performed by two authors independently, and potential conflicts were resolved with a third author. The Quality Assessment of Diagnostic Accuracy Studies (QUADAS-2) tool was used to evaluate the risk of bias in the primary diagnostic accuracy studies [15]. This tool consists of four domains concerning the selection of participants, the diagnostic test used, the gold standard evaluation, and the flow of the study, which are described in detail elsewhere. Authors assessed risk of bias in studies blinded to each other. Domains were assessed with either low, some concerns, or high risk of bias. The results of the quality assessment were displayed with a traffic plot graph. This was performed with robvis software [16].

Additionally, we applied a funnel plot to observe potential publication bias. However, the final judgement was done with Deek’s funnel asymmetry plot. P-value < 0.05 was evidence for presence of publication bias in the analysis.

## Results

### Screening results

**Fig. 1:**
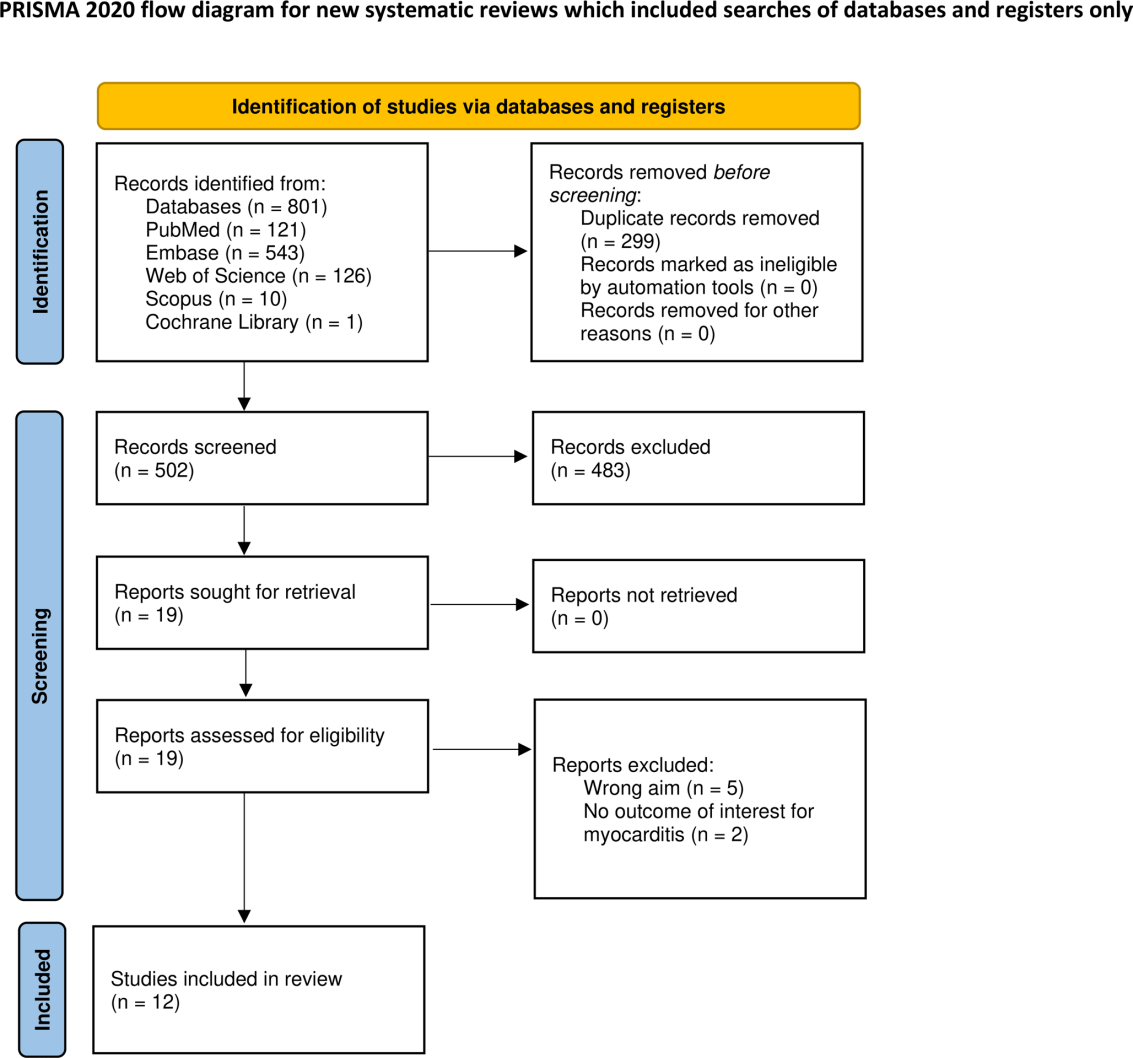
Screening process of the articles (flow diagram)

After comprehensive screening, this systematic review included 12 papers from the database search [17–28]—Fig. 1.

### Baseline demographic data and ML models summary

Half of the included studies were from Iran—Table 1. Majority of studies (10) assessed the performance of ML models on CMR imaging, while 2 papers assessed the performance on CMR reports.

**Table 1:** Characteristics of included studies

Study	Country	Assessment	Main model	Image size	Preprocessing and data augmentation	Pre-training	Data optimizer	Validation	Source of data
Zaman 2022	UK	Report (CMR)	BERT RL NLP	N/A	Anonymization, practitioner information removal, nonalphanumeric character removal, decapitalization, and white-space removal.	Pretrained on SciBERT on scientific publications, weighted binary cross-entropy loss function	Adam Optimizer	Split	London Hospitals
Zhu 2023	China	CMR	CNN with GAN, DE pre-training and RL	N/A	Online data augmentation with GAN-based model with random noise and actual data.	DE enhanced method, based on clustering mutation (k-means clustering) and updating scheme	N/A	5-fold cross	Z-Alizadeh Sani myocarditis dataset
Moravvej 2022	Iran	CMR	CNN with RL and ABC algorithm	100 × 100	Grayscale images with pixel intensity values normalized to a range between [0, 1]	Pretrained weights	ABC Optimizer	5-fold cross	Z-Alizadeh Sani myocarditis dataset
Yang 2023	Malaysia	CMR	Deep RL with improved differential evolution algorithm	100 × 100	Grayscale images with pixel intensity values normalized to a range between [0, 1]	Cluster-based mutation and update mechanism	DE algorithm	5-fold cross	Z-Alizadeh Sani myocarditis dataset
Shoeibi 2022	Iran	CMR	Resnet50d	N/A	Noise cancellation, and image resizing	CycleGAN pretrained	N/A	N/A	Z-Alizadeh Sani myocarditis dataset
Sharifrazi 2022	Iran	CMR	CNN-KCL 4-Cluster	100 × 100	Grayscale images with pixel intensity values normalized to a range between [0, 1]	N/A	N/A	10-fold cross	Z-Alizadeh Sani myocarditis dataset
Kasmae 2024	Iran	CMR	ELRL-DM	100 × 100	Grayscale images with pixel intensity values normalized to a range between [0, 1]	ABC pre-training, followed by deep Q-network training	N/A	5-fold cross	Z-Alizadeh Sani myocarditis dataset
Golilarz 2023	Iran	CMR	GAN-MD	100 × 100	GAN based online data augmentation, Grayscale images with pixel intensity values normalized to a range between [0, 1]	N/A	Focal loss training	5-fold cross	Z-Alizadeh Sani myocarditis dataset
Danaei 2022	Iran	CMR	CNN with ABC and RL	100 × 100	Grayscale images with pixel intensity values normalized to a range between [0, 1]	Imbalanced classification Markov decision process	ABC Optimizer	5-fold cross	Z-Alizadeh Sani myocarditis dataset
Noto 2019	Switzerland	CMR	SVM	N/A	Wavelet and exponential filters for noise reduction	N/A	N/A	10-fold cross	University Hospital Zurich
Kaya 2024	Germany	Report (CMR)	GPT-4	N/A	N/A	N/A	N/A	N/A	Eight tertiary care medical centers from Germany
Wang 2024a	China	CMR	Three ML - Video Swin Transformer (VST) with Long Short-Term Memory (LSTM) CNN and U-net-based deep neural network (DNN)	224 × 224	Rotations, scaling, gamma correction, mirroring, random rotation, random color jitter and adding random number	N/A	Adam optimizer and stochastic gradient descent (SGD) with Nesterov momentum (µ = 0.99)	3-fold cross	Beijing Fuwai Hospital
Wang 2024b								External	Beijing Anzhen Hospital, Guangdong Provincial People’s Hospital, the 2nd Affiliated Hospital of Harbin Medical University, the First Hospital of Lanzhou University, Renji Hospital, Tongji Hospital and Peking Union Medical College Hospital

Study	Country	Conditions in Dataset	Testing	Data type	Design of the study	Reference (labelling) of images	Myocarditis images in testing set	Training: Validation: Testing proportion	Training set size	Validation set size	Testing set size
Zaman 2022	UK	Myocarditis, Healthy, Dilated Cardiomyopathy, Hypertrophic Cardiomyopathy, and Myocardial Infarction	Internal	Experimental	Retrospective	Two clinicians with at least 3 years of CMR experience	N/A	53.3%:20%:26.6%	801	302	400
Zhu 2023	China	Myocarditis, Healthy	Internal	Experimental	Retrospective	N/A	N/A	N/A	N/A	N/A	N/A
Moravvej 2022	Iran	Myocarditis, Healthy	Internal	Experimental	Retrospective	N/A	4686	N/A	N/A	N/A	7135
Yang 2023	Malaysia	Myocarditis, Healthy	Internal	Experimental	Retrospective	N/A	4686	N/A	N/A	N/A	7135
Shoeibi 2022	Iran	Myocarditis, Healthy	Internal	Experimental	Retrospective	N/A	7000	N/A	N/A	N/A	13,000
Sharifrazi 2022	Iran	Myocarditis, Healthy	Internal	Experimental	Retrospective	N/A	61,334	N/A	N/A	N/A	98,898
Kasmae 2024	Iran	Myocarditis, Healthy	Internal	Experimental	Retrospective	N/A	4686	N/A	N/A	N/A	7135
Golilarz 2023	Iran	Myocarditis, Healthy	Internal	Experimental	Retrospective	N/A	4686	N/A	N/A	N/A	7135
Danaei 2022	Iran	Myocarditis, Healthy	Internal	Experimental	Retrospective	N/A	586	N/A	N/A	N/A	893
Noto 2019	Switzerland	Myocarditis, Myocardial Infarction	Internal	Experimental	Retrospective	Experts with more than 5 years of experience in CMR	62	81%:9%:10%	140	10	173 (average from 10-fold cross, each with 17 patients)
Kaya 2024	Germany	Myocarditis, No Myocarditis	Internal	Experimental	Retrospective	Two experts with 8 and 10 years of CMR experience	171	N/A	N/A	N/A	396
Wang 2024a	China	Hypertrophic Cardiomyopathy, Dilated Cardiomyopathy, Coronary Artery Disease, Left Ventricular Noncompaction Cardiomyopathy, Restrictive Cardiomyopathy, Cardiac Amyloidosis, Hypertensive Heart Disease, Myocarditis, Arrhythmogenic Right Ventricular Cardiomyopathy, Pulmonary Arterial Hypertension, Ebstein’s Anomaly, Healthy	Internal	Experimental	Retrospective	Group of CMR experts, conflicts resolved with seniors with > 15 years of CMR imaging experience	87	N/A	4433	N/A	6650
Wang 2024b			External				66	N/A	N/A	N/A	1416

Study	Country	Total dataset Size	Accuracy	Recall/Sensitivity	Specificity	Precision	F-score	AUC	MRI protocol	Hardware used for development	Software used for development
Zaman 2022	UK	1503	N/A	0.83	N/A	0.89	0.86	0.97 (95%CI 0.92-1.00)	N/A	NVIDIA P1000	Python 3
Zhu 2023	China	N/A	0.908 ± 0.020	0.886 ± 0.028	N/A	0.871 ± 0.040	0.878 ± 0.025	N/A	1.5-Tesla system with body coils in standard supine position. T1-weighted images in the axial views. After 10–15 min, late gadolinium enhancement were acquired in standard left ventricular short- and long-axis views. Protocols: CINE segmented (true FISP) long axis (LAX), TE 1.15 mm, TR 33.60 mm, NS 15, slice thickness 7 mm, concatenation and slice number 3, NE 1, breath hold time 8 s; CINE_segmented (true FISP) short axis, TE 1.11 mm, TR 31.92 mm, NS 15, slice thickness 7 mm, concatenation and slice number 15, NE 1, breath hold time 8 s; T2-weighted (TIRM) LAX, precontrast, TE 52 mm, TR 800 mm, slice thickness 10 mm, concatenation and slice number 3, NE 1, breath hold time 9 s; T2-weighted (TIRM) SAX, precontrast, TE 52 mm, TR 800 mm, slice thickness 10 mm, concatenation and slice number 5, NE 1, breath hold time 10 s; T1 relative-weighted TSE (Trigger)-AXIAdark blood pre- and postcontrast, TE 24 mm, TR 525 mm, slice thickness 8 mm, concatenation and slice number 5, NE 1, breath hold time 7 s; Late-GD enhancement LGE (high-resolution PSIR) SAX and LAX, TE 3.16 mm, TR 666 mm, slice thickness 8 mm, concatenation and slice number 1, NE 1, breath hold time 7 s;	N/A	N/A
Moravvej 2022	Iran	7135	0.886 ± 0.02	0.863 ± 0.017	0.901 ± 0.024	0.84 ± 0.034	0.851 ± 0.024	N/A		N/A	Python, PyTorch
Yang 2023	Malaysia	7135	0.910 ± 0.019	0.889 ± 0.038	0.926 ± 0.021	0.875 ± 0.043	0.883 ± 0.029	N/A		N/A	Python, PyTorch
Shoeibi 2022	Iran	13,000	0.9954 ± 0.0258	0.9947 ± 0.2046	0.9957 ± 0.0689	0.9919 ± 0.1564	0.9933 ± 0.212	N/A		16 GB RAM, CPU Core i7, and NVidia GeForce 1080	Python environment using the TensorFlow 2, Keras, and Skit-learn
Sharifrazi 2022	Iran	98,898	0.9741	0.957	0.9856	0.976	0.965	0.9705		N/A	Python, Tensorflow
Kasmae 2024	Iran	7135	0.911 ± 0.019	0.888 ± 0.018	0.925 ± 0.025	0.877 ± 0.038	0.882 ± 0.023	N/A		N/A	N/A
Golilarz 2023	Iran	7135	0.912 ± 0.03	0.898 ± 0.018	0.931 ± 0.032	0.883 ± 0.051	0.862 ± 0.032	N/A		64 GB RAM	Python, PyTorch
Danaei 2022	Iran	893	0.895 ± 0.018	0.871 ± 0.022	0.91 ± 0.025	0.854 ± 0.036	0.862 ± 0.022	N/A		N/A	Python, PyTorch
Noto 2019	Switzerland	173	0.89 ± 0.01	0.93 ± 0.01	0.82 ± 0.04	0.9 ± 0.02	N/A	N/A	1.5-T scanner (Achieva; Philips Healthcare, Best, the Netherlands) x162; 3-T scanner (MAGNETOM Skyra; Siemens Healthcare, Erlangen, Germany) x11. 5-element and 18-element phased-array receiver coils. Cine images acquired in short-axis, two-chamber, three-chamber, and four-chamber views. T2-weighted double-inversion black-blood spin-echo sequence in three short-axis slices. LGE images in three short-axis slices acquired 10–15 min after administration of a bolus. A 3D inversion recovery gradient-echo MRI sequence for 1.5 T and 2D phase-sensitive inversion recovery sequence for 3 T were used. 512 × 512 matrix images	N/A	N/A
Kaya 2024	Germany	396	0.83	0.9	0.78	0.76	0.82	N/A	N/A	N/A	OpenAI GPT-4 Chat
Wang 2024a	China	6650	N/A	0.966 (95% CI 0.923-1.000)	0.971 (95% CI 0.922–0.993)	N/A	0.724 (95% CI 0.638–0.795)	0.987 (95% CI 0.978–0.995)	Twenty different imaging protocols were used with SIEMENS, GE Healthcare, and Philips hardware. Magnetic field strength of 3 was used in all protocols. Slice thickness was 8 mm (19/20) and 10 mm (1/20). Slice spacing varied between 6 to 10 mm. Field of view ranged from 24 to 38 cm. Echo time ranged from 1.20 to 3.00 ms. Repetition time ranged from 5.98 to 6.13 ms. Inversion time ranged from 280 to 375 ms. Temportal resolution ranged from 37.68 to 80.00 ms. Flip angle varied between 20 to 55 degrees. Pixel Bandwidth ranged from 226 to 2188 Hz/pixel.	Four NVIDIA GeForce RTX 3090 24 GB VRAM	N/A
Wang 2024b		1416		0.955 (95% CI 0.855-1.000)	0.955 (95% CI 0.885-1.000)		0.630 (95% CI 0.514–0.735)	0.972 (95% CI 0.95–0.989)			

In this systematic review, we have synthesized a total of 141 unique results of ML-based CMR diagnosis of myocarditis from 12 studies. These models included CNN models (e.g., efficientnet b3, efficientnetv2, resnest50d, resnet50d, resnetrs50, AlexNET, GoogleNET, DenseNet, MobileNet), SVMs, k-nearest neighbor algorithms (KNNs), naive Bayes, random forest (RF), logistic regression, and GPT-4 based chat. The full list is available in Table 2, as many studies used custom modified CNNs.

**Table 2:** Diagnostic accuracy of various ML models from the studies

Study	Algorithm	Testing Set Conditions	Total number of conditions	Data type	Testing set (*n*)
Zhu 2023	CNN with RL	Healthy, Myocarditis	2	CMR	N/A
Zhu 2023	CNN with RL with GAN	Healthy, Myocarditis	2	CMR	N/A
Zhu 2023	GDA	Healthy, Myocarditis	2	CMR	N/A
Zhu 2023	OSS	Healthy, Myocarditis	2	CMR	N/A
Zhu 2023	BR	Healthy, Myocarditis	2	CMR	N/A
Zhu 2023	BAT	Healthy, Myocarditis	2	CMR	N/A
Zhu 2023	COA	Healthy, Myocarditis	2	CMR	N/A
Zhu 2023	DE	Healthy, Myocarditis	2	CMR	N/A
Zhu 2023	AlexNet	Healthy, Myocarditis	2	CMR	N/A
Zhu 2023	GoogleNet	Healthy, Myocarditis	2	CMR	N/A
Zhu 2023	ResNet	Healthy, Myocarditis	2	CMR	N/A
Zhu 2023	DenseNet	Healthy, Myocarditis	2	CMR	N/A
Zhu 2023	MobileNet	Healthy, Myocarditis	2	CMR	N/A
Zhu 2023	CNN with loss function WCE	Healthy, Myocarditis	2	CMR	N/A
Zhu 2023	CNN with loss function BCE	Healthy, Myocarditis	2	CMR	N/A
Zhu 2023	CNN with loss function DL	Healthy, Myocarditis	2	CMR	N/A
Zhu 2023	CNN with loss function TL	Healthy, Myocarditis	2	CMR	N/A
Zaman 2022	BERT NLP	Healthy, Myocarditis + 3 CVD	5	CMR reports	400
Zaman 2022	SVM	Healthy, Myocarditis + 3 CVD	5	CMR reports	400
Moravvej 2022	CNN-KCL	Healthy, Myocarditis	2	CMR	7135
Moravvej 2022	CNN random weight	Healthy, Myocarditis	2	CMR	7135
Moravvej 2022	CNN ABC	Healthy, Myocarditis	2	CMR	7135
Moravvej 2022	CNN RL	Healthy, Myocarditis	2	CMR	7135
Moravvej 2022	RLMD-PA CNN + ABC + RL	Healthy, Myocarditis	2	CMR	7135
Moravvej 2022	SVM	Healthy, Myocarditis	2	CMR	7135
Moravvej 2022	KNN	Healthy, Myocarditis	2	CMR	7135
Moravvej 2022	Naive Bayes	Healthy, Myocarditis	2	CMR	7135
Moravvej 2022	Logistic Regression	Healthy, Myocarditis	2	CMR	7135
Moravvej 2022	RF	Healthy, Myocarditis	2	CMR	7135
Moravvej 2022	CNN + GDM + RL	Healthy, Myocarditis	2	CMR	7135
Moravvej 2022	CNN + GDA + RL	Healthy, Myocarditis	2	CMR	7135
Moravvej 2022	CNN + GDMA + RL	Healthy, Myocarditis	2	CMR	7135
Moravvej 2022	CNN + OSS + RL	Healthy, Myocarditis	2	CMR	7135
Moravvej 2022	CNN + BR + RL	Healthy, Myocarditis	2	CMR	7135
Moravvej 2022	CNN + GWO + RL	Healthy, Myocarditis	2	CMR	7135
Moravvej 2022	CNN + BAT + RL	Healthy, Myocarditis	2	CMR	7135
Moravvej 2022	CNN + COA + RL	Healthy, Myocarditis	2	CMR	7135
Moravvej 2022	CNN + WOA + RL	Healthy, Myocarditis	2	CMR	7135
Yang 2023	CNN-KCL	Healthy, Myocarditis	2	CMR	7135
Yang 2023	RLMD-PA	Healthy, Myocarditis	2	CMR	7135
Yang 2023	SVM	Healthy, Myocarditis	2	CMR	7135
Yang 2023	KNN	Healthy, Myocarditis	2	CMR	7135
Yang 2023	Naïve Bayes	Healthy, Myocarditis	2	CMR	7135
Yang 2023	Logistic Regression	Healthy, Myocarditis	2	CMR	7135
Yang 2023	Random Forests	Healthy, Myocarditis	2	CMR	7135
Yang 2023	MD + random weight	Healthy, Myocarditis	2	CMR	7135
Yang 2023	MD + IDE	Healthy, Myocarditis	2	CMR	7135
Yang 2023	MD + RL	Healthy, Myocarditis	2	CMR	7135
Yang 2023	IDE + RL	Healthy, Myocarditis	2	CMR	7135
Yang 2023	GDM	Healthy, Myocarditis	2	CMR	7135
Yang 2023	GDMA	Healthy, Myocarditis	2	CMR	7135
Yang 2023	GWO	Healthy, Myocarditis	2	CMR	7135
Yang 2023	WCE	Healthy, Myocarditis	2	CMR	7135
Yang 2023	BCE	Healthy, Myocarditis	2	CMR	7135
Yang 2023	FL	Healthy, Myocarditis	2	CMR	7135
Yang 2023	DE	Healthy, Myocarditis	2	CMR	7135
Yang 2023	TL	Healthy, Myocarditis	2	CMR	7135
Shoeibi 2022	efficientnet b3	Healthy, Myocarditis	2	CMR	13,000
Shoeibi 2022	efficientnetv2	Healthy, Myocarditis	2	CMR	13,000
Shoeibi 2022	HrNet	Healthy, Myocarditis	2	CMR	13,000
Shoeibi 2022	resnest50d	Healthy, Myocarditis	2	CMR	13,000
Shoeibi 2022	resnet50d	Healthy, Myocarditis	2	CMR	13,000
Shoeibi 2022	resnetrs50	Healthy, Myocarditis	2	CMR	13,000
Shoeibi 2022	efficientnet GAN	Healthy, Myocarditis	2	CMR	13,000
Shoeibi 2022	efficientnetv2 GAN	Healthy, Myocarditis	2	CMR	13,000
Shoeibi 2022	HrNet GAN	Healthy, Myocarditis	2	CMR	13,000
Shoeibi 2022	resnest50d GAN	Healthy, Myocarditis	2	CMR	13,000
Shoeibi 2022	resnet50d GAN	Healthy, Myocarditis	2	CMR	13,000
Shoeibi 2022	resnetrs50 GAN	Healthy, Myocarditis	2	CMR	13,000
Sharifrazi 2022	CNN-KCL 2-Cluster	Healthy, Myocarditis	2	CMR	98,898
Sharifrazi 2022	CNN-KCL 4-Cluster	Healthy, Myocarditis	2	CMR	98,898
Sharifrazi 2022	CNN-KCL 10-Cluster	Healthy, Myocarditis	2	CMR	98,898
Sharifrazi 2022	CNN-KCL 24-Cluster	Healthy, Myocarditis	2	CMR	98,898
Sharifrazi 2022	BN	Healthy, Myocarditis	2	CMR	98,898
Sharifrazi 2022	DT	Healthy, Myocarditis	2	CMR	98,898
Sharifrazi 2022	LR	Healthy, Myocarditis	2	CMR	98,898
Sharifrazi 2022	RF	Healthy, Myocarditis	2	CMR	98,898
Sharifrazi 2022	CNN-KCL	Healthy, Myocarditis	2	CMR	98,898
Kasmae 2024	CNN-KCL	Healthy, Myocarditis	2	CMR	7135
Kasmae 2024	3-CNN + ABC	Healthy, Myocarditis	2	CMR	7135
Kasmae 2024	3-CNN + RL	Healthy, Myocarditis	2	CMR	7135
Kasmae 2024	ELRL-DM	Healthy, Myocarditis	2	CMR	7135
Kasmae 2024	SVM	Healthy, Myocarditis	2	CMR	7135
Kasmae 2024	KNN	Healthy, Myocarditis	2	CMR	7135
Kasmae 2024	Naïve Bayes	Healthy, Myocarditis	2	CMR	7135
Kasmae 2024	Logistic Regression	Healthy, Myocarditis	2	CMR	7135
Kasmae 2024	RF	Healthy, Myocarditis	2	CMR	7135
Kasmae 2024	3-CNN + ABS + RL	Healthy, Myocarditis	2	CMR	7135
Kasmae 2024	3-CNN + GWO + RL	Healthy, Myocarditis	2	CMR	7135
Kasmae 2024	3-CNN + FA + RL	Healthy, Myocarditis	2	CMR	7135
Kasmae 2024	3-CNN + WOA + RL	Healthy, Myocarditis	2	CMR	7135
Kasmae 2024	3-CNN + COA + RL	Healthy, Myocarditis	2	CMR	7135
Kasmae 2024	3-CNN + HMS + RL	Healthy, Myocarditis	2	CMR	7135
Kasmae 2024	3-CNN + BAT + RL	Healthy, Myocarditis	2	CMR	7135
Kasmae 2024	3-CNN + SSA + RL	Healthy, Myocarditis	2	CMR	7135
Kasmae 2024	3-CNN + DE + RL	Healthy, Myocarditis	2	CMR	7135
Kasmae 2024	AlexNet	Healthy, Myocarditis	2	CMR	7135
Kasmae 2024	GoogleNet	Healthy, Myocarditis	2	CMR	7135
Kasmae 2024	ResNet	Healthy, Myocarditis	2	CMR	7135
Kasmae 2024	DenseNet	Healthy, Myocarditis	2	CMR	7135
Kasmae 2024	MobileNet	Healthy, Myocarditis	2	CMR	7135
Golilarz 2023	CNN-KCL	Healthy, Myocarditis	2	CMR	7135
Golilarz 2023	RLMD-PA	Healthy, Myocarditis	2	CMR	7135
Golilarz 2023	MD	Healthy, Myocarditis	2	CMR	7135
Golilarz 2023	GAN-MD	Healthy, Myocarditis	2	CMR	7135
Golilarz 2023	AlexNet	Healthy, Myocarditis	2	CMR	7135
Golilarz 2023	GoogleNet	Healthy, Myocarditis	2	CMR	7135
Golilarz 2023	ResNet	Healthy, Myocarditis	2	CMR	7135
Golilarz 2023	DenseNet	Healthy, Myocarditis	2	CMR	7135
Golilarz 2023	MobileNet	Healthy, Myocarditis	2	CMR	7135
Golilarz 2023	WCE	Healthy, Myocarditis	2	CMR	7135
Golilarz 2023	BCE	Healthy, Myocarditis	2	CMR	7135
Golilarz 2023	DL	Healthy, Myocarditis	2	CMR	7135
Golilarz 2023	TL	Healthy, Myocarditis	2	CMR	7135
Golilarz 2023	GAN	Healthy, Myocarditis	2	CMR	7135
Golilarz 2023	DRAGAN	Healthy, Myocarditis	2	CMR	7135
Golilarz 2023	AGE	Healthy, Myocarditis	2	CMR	7135
Golilarz 2023	α-GAN	Healthy, Myocarditis	2	CMR	7135
Danaei 2022	CNN-KCL	Healthy, Myocarditis	2	CMR	893
Danaei 2022	RLMD-PA	Healthy, Myocarditis	2	CMR	893
Danaei 2022	CNN + BA + RL	Healthy, Myocarditis	2	CMR	893
Danaei 2022	CNN + DE + RL	Healthy, Myocarditis	2	CMR	893
Danaei 2022	CNN + ABC + RL	Healthy, Myocarditis	2	CMR	893
Danaei 2022	WCE	Healthy, Myocarditis	2	CMR	893
Danaei 2022	BCE	Healthy, Myocarditis	2	CMR	893
Danaei 2022	FL	Healthy, Myocarditis	2	CMR	893
Danaei 2022	DE	Healthy, Myocarditis	2	CMR	893
Danaei 2022	TL	Healthy, Myocarditis	2	CMR	893
Noto 2019	KNN (2D)	MI, Myocarditis	2	CMR	173
Noto 2019	LDA (2D)	MI, Myocarditis	2	CMR	173
Noto 2019	Multilayer perceptron NN (2D)	MI, Myocarditis	2	CMR	173
Noto 2019	SVM (2D)	MI, Myocarditis	2	CMR	173
Noto 2019	TB (RF) (2D)	MI, Myocarditis	2	CMR	173
Noto 2019	KNN (3D)	MI, Myocarditis	2	CMR	173
Noto 2019	LDA (3D)	MI, Myocarditis	2	CMR	173
Noto 2019	Multilayer perceptron NN (3D)	MI, Myocarditis	2	CMR	173
Noto 2019	SVM (3D)	MI, Myocarditis	2	CMR	173
Noto 2019	TB (RF) (3D)	MI, Myocarditis	2	CMR	173
Kaya 2024	GPT-4	Myocarditis, No Myocarditis	2	CMR report	396
Wang 2024a	Three ML - Internal	Multiple	12	CMR	6650
Wang 2024b	Three ML - External	Multiple	12	CMR	1416

Study	Algorithm	Myocarditis in testing set (*n*)	Sensitivity	Specificity	Precision	Accuracy	F-Score	G-Means	AUC
Zhu 2023	CNN with RL	N/A	0.826 ± 0.027	N/A	0.804 ± 0.038	0.858 ± 0.023	0.814 ± 0.027	N/A	N/A
Zhu 2023	CNN with RL with GAN	N/A	0.886 ± 0.028	N/A	0.871 ± 0.040	0.908 ± 0.020	0.878 ± 0.025	N/A	N/A
Zhu 2023	GDA	N/A	0.811 ± 0.024	N/A	0.781 ± 0.043	0.841 ± 0.019	0.794 ± 0.021	N/A	N/A
Zhu 2023	OSS	N/A	0.800 ± 0.023	N/A	0.794 ± 0.021	0.845 ± 0.011	0.797 ± 0.026	N/A	N/A
Zhu 2023	BR	N/A	0.783 ± 0.010	N/A	0.780 ± 0.042	0.833 ± 0.010	0.786 ± 0.017	N/A	N/A
Zhu 2023	BAT	N/A	0.798 ± 0.029	N/A	0.810 ± 0.019	0.855 ± 0.021	0.801 ± 0.018	N/A	N/A
Zhu 2023	COA	N/A	0.806 ± 0.006	N/A	0.789 ± 0.032	0.849 ± 0.010	0.790 ± 0.045	N/A	N/A
Zhu 2023	DE	N/A	0.871 ± 0.020	N/A	0.852 ± 0.031	0.893 ± 0.014	0.870 ± 0.019	N/A	N/A
Zhu 2023	AlexNet	N/A	0.682	N/A	0.618	0.761	0.625	N/A	N/A
Zhu 2023	GoogleNet	N/A	0.764	N/A	0.651	0.752	0.707	N/A	N/A
Zhu 2023	ResNet	N/A	0.706	N/A	0.645	0.743	0.659	N/A	N/A
Zhu 2023	DenseNet	N/A	0.720	N/A	0.622	0.732	0.659	N/A	N/A
Zhu 2023	MobileNet	N/A	0.734	N/A	0.652	0.763	0.706	N/A	N/A
Zhu 2023	CNN with loss function WCE	N/A	0.732	N/A	0.758	0.801	0.744	N/A	N/A
Zhu 2023	CNN with loss function BCE	N/A	0.805	N/A	0.714	0.800	0.745	N/A	N/A
Zhu 2023	CNN with loss function DL	N/A	0.795	N/A	0.701	0.806	0.715	N/A	N/A
Zhu 2023	CNN with loss function TL	N/A	0.774	N/A	0.710	0.813	0.752	N/A	N/A
Zaman 2022	BERT NLP	29	0.83	N/A	0.89	N/A	0.86	N/A	0.97 (0.92-1.00)
Zaman 2022	SVM	29	0.76	N/A	0.65	N/A	0.7	N/A	N/A
Moravvej 2022	CNN-KCL	4686	0.751 ± 0.032	0.845 ± 0.022	0.745 ± 0.032	0.81 ± 0.024	0.748 ± 0.031	0.797 ± 0.025	N/A
Moravvej 2022	CNN random weight	4686	0.717 ± 0.213	0.806 ± 0.02	0.691 ± 0.029	0.772 ± 0.021	0.704 ± 0.026	0.76 ± 0.021	N/A
Moravvej 2022	CNN ABC	4686	0.771 ± 0.027	0.842 ± 0.018	0.746 ± 0.027	0.815 ± 0.02	0.758 ± 0.026	0.806 ± 0.021	N/A
Moravvej 2022	CNN RL	4686	0.801 ± 0.028	0.863 ± 0.02	0.779 ± 0.029	0.84 ± 0.021	0.79 ± 0.026	0.831 ± 0.022	N/A
Moravvej 2022	RLMD-PA CNN + ABC + RL	4686	0.863 ± 0.017	0.901 ± 0.024	0.84 ± 0.034	0.886 ± 0.02	0.851 ± 0.024	0.882 ± 0.019	N/A
Moravvej 2022	SVM	4686	0.737 ± 0.042	0.651 ± 0.093	0.565 ± 0.074	0.683 ± 0.07	0.639 ± 0.06	0.692 ± 0.065	N/A
Moravvej 2022	KNN	4686	0.589 ± 0.111	0.587 ± 0.039	0.46 ± 0.072	0.588 ± 0.064	0.516 ± 0.089	0.587 ± 0.075	N/A
Moravvej 2022	Naive Bayes	4686	0.565 ± 0.134	0.645 ± 0.031	0.484 ± 0.062	0.615 ± 0.051	0.52 ± 0.092	0.6 ± 0.072	N/A
Moravvej 2022	Logistic Regression	4686	0.657 ± 0.057	0.663 ± 0.049	0.541 ± 0.041	0.661 ± 0.038	0.593 ± 0.042	0.659 ± 0.038	N/A
Moravvej 2022	RF	4686	0.648 ± 0.071	0.459 ± 0.071	0.42 ± 0.056	0.53 ± 0.07	0.509 ± 0.063	0.545 ± 0.071	N/A
Moravvej 2022	CNN + GDM + RL	4686	0.806 ± 0.018	0.875 ± 0.028	0.796 ± 0.038	0.849 ± 0.022	0.801 ± 0.026	0.84 ± 0.021	N/A
Moravvej 2022	CNN + GDA + RL	4686	0.808 ± 0.022	0.86 ± 0.028	0.778 ± 0.035	0.84 ± 0.017	0.792 ± 0.019	0.834 ± 0.015	N/A
Moravvej 2022	CNN + GDMA + RL	4686	0.817 ± 0.037	0.877 ± 0.026	0.8 ± 0.037	0.854 ± 0.025	0.808 ± 0.033	0.846 ± 0.026	N/A
Moravvej 2022	CNN + OSS + RL	4686	0.804 ± 0.037	0.872 ± 0.01	0.791 ± 0.015	0.846 ± 0.016	0.797 ± 0.024	0.837 ± 0.021	N/A
Moravvej 2022	CNN + BR + RL	4686	0.785 ± 0.027	0.868 ± 0.034	0.784 ± 0.041	0.837 ± 0.012	0.784 ± 0.007	0.825 ± 0.003	N/A
Moravvej 2022	CNN + GWO + RL	4686	0.804 ± 0.027	0.877 ± 0.013	0.797 ± 0.02	0.85 ± 0.016	0.801 ± 0.021	0.84 ± 0.018	N/A
Moravvej 2022	CNN + BAT + RL	4686	0.796 ± 0.024	0.885 ± 0.01	0.807 ± 0.016	0.851 ± 0.013	0.801 ± 0.018	0.839 ± 0.016	N/A
Moravvej 2022	CNN + COA + RL	4686	0.813 ± 0.046	0.862 ± 0.028	0.781 ± 0.039	0.844 ± 0.028	0.796 ± 0.038	0.837 ± 0.031	N/A
Moravvej 2022	CNN + WOA + RL	4686	0.789 ± 0.021	0.866 ± 0.021	0.781 ± 0.024	0.837 ± 0.012	0.785 ± 0.014	0.827 ± 0.012	N/A
Yang 2023	CNN-KCL	4686	0.744 ± 0.033	0.838 ± 0.027	0.735 ± 0.036	0.804 ± 0.015	0.740 ± 0.030	0.792 ± 0.028	N/A
Yang 2023	RLMD-PA	4686	0.845 ± 0.016	0.896 ± 0.026	0.829 ± 0.035	0.878 ± 0.019	0.837 ± 0.017	0.869 ± 0.015	N/A
Yang 2023	SVM	4686	0.786 ± 0.029	0.692 ± 0.020	0.606 ± 0.024	0.727 ± 0.023	0.684 ± 0.026	0.737 ± 0.023	N/A
Yang 2023	KNN	4686	0.706 ± 0.020	0.724 ± 0.150	0.631 ± 0.151	0.718 ± 0.100	0.660 ± 0.090	0.713 ± 0.082	N/A
Yang 2023	Naïve Bayes	4686	0.782 ± 0.039	0.649 ± 0.013	0.572 ± 0.016	0.699 ± 0.017	0.661 ± 0.023	0.712 ± 0.020	N/A
Yang 2023	Logistic Regression	4686	0.679 ± 0.015	0.659 ± 0.018	0.545 ± 0.018	0.666 ± 0.016	0.605 ± 0.017	0.669 ± 0.016	N/A
Yang 2023	Random Forests	4686	0.665 ± 0.033	0.531 ± 0.022	0.461 ± 0.020	0.582 ± 0.022	0.544 ± 0.024	0.594 ± 0.022	N/A
Yang 2023	MD + random weight	4686	0.697 ± 0.045	0.801 ± 0.031	0.679 ± 0.047	0.762 ± 0.035	0.687 ± 0.046	0.747 ± 0.038	N/A
Yang 2023	MD + IDE	4686	0.864 ± 0.044	0.885 ± 0.016	0.832 ± 0.040	0.871 ± 0.032	0.838 ± 0.031	0.876 ± 0.030	N/A
Yang 2023	MD + RL	4686	0.870 ± 0.042	0.910 ± 0.019	0.851 ± 0.049	0.893 ± 0.025	0.860 ± 0.032	0.896 ± 0.034	N/A
Yang 2023	IDE + RL	4686	0.889 ± 0.038	0.926 ± 0.021	0.875 ± 0.043	0.910 ± 0.019	0.883 ± 0.029	0.919 ± 0.027	N/A
Yang 2023	GDM	4686	0.804 ± 0.019	0.878 ± 0.029	0.793 ± 0.039	0.829 ± 0.021	0.804 ± 0.036	0.841 ± 0.024	N/A
Yang 2023	GDMA	4686	0.810 ± 0.032	0.879 ± 0.028	0.804 ± 0.030	0.857 ± 0.014	0.809 ± 0.035	0.849 ± 0.028	N/A
Yang 2023	GWO	4686	0.802 ± 0.023	0.879 ± 0.016	0.799 ± 0.024	0.856 ± 0.019	0.801 ± 0.024	0.842 ± 0.019	N/A
Yang 2023	WCE	4686	0.778 ± 0.013	0.885 ± 0.021	0.803 ± 0.021	0.845 ± 0.003	0.790 ± 0.005	0.830 ± 0.015	N/A
Yang 2023	BCE	4686	0.824 ± 0.024	0.821 ± 0.035	0.745 ± 0.000	0.822 ± 0.001	0.783 ± 0.031	0.822 ± 0.006	N/A
Yang 2023	FL	4686	0.833 ± 0.027	0.889 ± 0.036	0.819 ± 0.006	0.868 ± 0.026	0.826 ± 0.027	0.861 ± 0.026	N/A
Yang 2023	DE	4686	0.837 ± 0.026	0.795 ± 0.001	0.711 ± 0.009	0.811 ± 0.024	0.769 ± 0.031	0.816 ± 0.033	N/A
Yang 2023	TL	4686	0.816 ± 0.020	0.834 ± 0.026	0.747 ± 0.007	0.827 ± 0.013	0.780 ± 0.010	0.825 ± 0.024	N/A
Shoeibi 2022	efficientnet b3	7000	0.9914 ± 0.3096	0.9972 ± 0.1785	0.9947 ± 0.1787	0.9952 ± 0.0348	0.993 ± 0.2407	N/A	N/A
Shoeibi 2022	efficientnetv2	7000	0.9947 ± 0.2046	0.9957 ± 0.0689	0.9919 ± 0.1564	0.9954 ± 0.0258	0.9933 ± 0.212	N/A	N/A
Shoeibi 2022	HrNet	7000	0.9912 ± 0.0051	0.99 ± 0.0016	0.9811 ± 0.0031	0.9904 ± 0.0028	0.9861 ± 0.0041	N/A	N/A
Shoeibi 2022	resnest50d	7000	0.9806 ± 0.0072	0.9984 ± 0.0023	0.9969 ± 0.0044	0.9923 ± 0.004	0.9887 ± 0.0058	N/A	N/A
Shoeibi 2022	resnet50d	7000	0.9939 ± 0.0038	0.9974 ± 0.0014	0.9951 ± 0.0027	0.9962 ± 0.0014	0.9945 ± 0.002	N/A	N/A
Shoeibi 2022	resnetrs50	7000	0.9929 ± 0.0103	0.9949 ± 0.0037	0.9904 ± 0.0069	0.9942 ± 0.0033	0.9916 ± 0.0048	N/A	N/A
Shoeibi 2022	efficientnet GAN	7000	0.9857 ± 0.0142	0.9972 ± 0.0018	0.9947 ± 0.0034	0.9933 ± 0.0037	0.9901 ± 0.0056	N/A	N/A
Shoeibi 2022	efficientnetv2 GAN	7000	0.9903 ± 0.0087	0.9949 ± 0.0037	0.9904 ± 0.007	0.9933 ± 0.0007	0.9903 ± 0.0011	N/A	N/A
Shoeibi 2022	HrNet GAN	7000	0.8947 ± 0.0988	0.8434 ± 0.2245	0.8166 ± 0.2132	0.861 ± 0.1325	0.8333 ± 0.1212	N/A	N/A
Shoeibi 2022	resnest50d GAN	7000	0.9816 ± 0.0195	0.9921 ± 0.0029	0.9849 ± 0.0054	0.9885 ± 0.0068	0.9832 ± 0.0101	N/A	N/A
Shoeibi 2022	resnet50d GAN	7000	0.9898 ± 0.005	0.9942 ± 0.0042	0.9891 ± 0.0078	0.9927 ± 0.002	0.9894 ± 0.0029	N/A	N/A
Shoeibi 2022	resnetrs50 GAN	7000	0.9931 ± 0,007	0.9846 ± 0.0103	0.9716 ± 0.0184	0.9875 ± 0.0061	0.9821 ± 0.0086	N/A	N/A
Sharifrazi 2022	CNN-KCL 2-Cluster	61,334	0.927	0.9678	0.946	0.9524	0.938	N/A	0.9475
Sharifrazi 2022	CNN-KCL 4-Cluster	61,334	0.957	0.9856	0.976	0.9741	0.965	N/A	0.9705
Sharifrazi 2022	CNN-KCL 10-Cluster	61,334	0.945	0.9871	0.978	0.9704	0.959	N/A	0.9651
Sharifrazi 2022	CNN-KCL 24-Cluster	61,334	0.937	0.99	0.983	0.9698	0.961	N/A	0.9634
Sharifrazi 2022	BN	61,334	0.773	0.3994	0.44	0.5411	0.562	N/A	0.5859
Sharifrazi 2022	DT	61,334	0.9	0.9337	0.891	0.9199	0.894	N/A	0.9155
Sharifrazi 2022	LR	61,334	0.851	0.922	0.869	0.8952	0.861	N/A	0.8868
Sharifrazi 2022	RF	61,334	0.923	0.957	0.929	0.944	0.927	N/A	0.94
Sharifrazi 2022	CNN-KCL	61,334	0.957	0.9856	0.976	0.9741	0.965	N/A	0.9705
Kasmae 2024	CNN-KCL	4686	0.782 ± 0.027	0.863 ± 0.034	0.775 ± 0.048	0.832 ± 0.03	0.779 ± 0.037	0.821 ± 0.029	N/A
Kasmae 2024	3-CNN + ABC	4686	0.765 ± 0.041	0.849 ± 0.025	0.753 ± 0.04	0.817 ± 0.03	0.759 ± 0.04	0.806 ± 0.032	N/A
Kasmae 2024	3-CNN + RL	4686	0.808 ± 0.032	0.873 ± 0.023	0.794 ± 0.034	0.849 ± 0.021	0.801 ± 0.028	0.84 ± 0.023	N/A
Kasmae 2024	ELRL-DM	4686	0.888 ± 0.018	0.925 ± 0.025	0.877 ± 0.038	0.911 ± 0.019	0.882 ± 0.023	0.906 ± 0.017	N/A
Kasmae 2024	SVM	4686	0.79 ± 0.037	0.7 ± 0.027	0.614 ± 0.032	0.734 ± 0.03	0.691 ± 0.034	0.744 ± 0.031	N/A
Kasmae 2024	KNN	4686	0.709 ± 0.03	0.727 ± 0.151	0.634 ± 0.156	0.721 ± 0.104	0.664 ± 0.097	0.716 ± 0.087	N/A
Kasmae 2024	Naïve Bayes	4686	0.787 ± 0.049	0.646 ± 0.016	0.572 ± 0.015	0.699 ± 0.017	0.662 ± 0.026	0.713 ± 0.021	N/A
Kasmae 2024	Logistic Regression	4686	0.692 ± 0.026	0.664 ± 0.026	0.554 ± 0.028	0.675 ± 0.025	0.615 ± 0.027	0.678 ± 0.025	N/A
Kasmae 2024	RF	4686	0.672 ± 0.034	0.526 ± 0.035	0.461 ± 0.029	0.581 ± 0.032	0.546 ± 0.031	0.594 ± 0.032	N/A
Kasmae 2024	3-CNN + ABS + RL	4686	0.81 ± 0.013	0.877 ± 0.016	0.799 ± 0.023	0.852 ± 0.015	0.804 ± 0.017	0.843 ± 0.014	N/A
Kasmae 2024	3-CNN + GWO + RL	4686	0.807 ± 0.024	0.856 ± 0.006	0.771 ± 0.009	0.837 ± 0.009	0.788 ± 0.014	0.831 ± 0.013	N/A
Kasmae 2024	3-CNN + FA + RL	4686	0.781 ± 0.014	0.876 ± 0.016	0.791 ± 0.024	0.84 ± 0.014	0.786 ± 0.018	0.827 ± 0.014	N/A
Kasmae 2024	3-CNN + WOA + RL	4686	0.74 ± 0.021	0.868 ± 0.009	0.771 ± 0.011	0.82 ± 0.008	0.755 ± 0.013	0.801 ± 0.011	N/A
Kasmae 2024	3-CNN + COA + RL	4686	0.807 ± 0.024	0.885 ± 0.008	0.809 ± 0.013	0.856 ± 0.012	0.808 ± 0.017	0.845 ± 0.014	N/A
Kasmae 2024	3-CNN + HMS + RL	4686	0.793 ± 0.019	0.866 ± 0.01	0.781 ± 0.014	0.838 ± 0.011	0.787 ± 0.014	0.828 ± 0.012	N/A
Kasmae 2024	3-CNN + BAT + RL	4686	0.808 ± 0.015	0.883 ± 0.012	0.806 ± 0.018	0.854 ± 0.012	0.807 ± 0.015	0.845 ± 0.012	N/A
Kasmae 2024	3-CNN + SSA + RL	4686	0.771 ± 0.013	0.864 ± 0.017	0.773 ± 0.021	0.829 ± 0.012	0.772 ± 0.014	0.816 ± 0.011	N/A
Kasmae 2024	3-CNN + DE + RL	4686	0.838 ± 0.006	0.904 ± 0.022	0.841 ± 0.032	0.879 ± 0.015	0.839 ± 0.017	0.87 ± 0.012	N/A
Kasmae 2024	AlexNet	4686	0.715 ± 0.029	0.836 ± 0.034	0.724 ± 0.034	0.79 ± 0.01	0.719 ± 0.015	0.773 ± 0.012	N/A
Kasmae 2024	GoogleNet	4686	0.811 ± 0.026	0.768 ± 0.035	0.676 ± 0.015	0.784 ± 0.014	0.737 ± 0.045	0.789 ± 0.021	N/A
Kasmae 2024	ResNet	4686	0.736 ± 0.032	0.794 ± 0.023	0.683 ± 0.014	0.772 ± 0.027	0.709 ± 0.019	0.764 ± 0.003	N/A
Kasmae 2024	DenseNet	4686	0.748 ± 0.018	0.759 ± 0.033	0.651 ± 0.029	0.755 ± 0.015	0.696 ± 0.025	0.753 ± 0.01	N/A
Kasmae 2024	MobileNet	4686	0.762 ± 0.046	0.806 ± 0.003	0.703 ± 0.02	0.789 ± 0.024	0.731 ± 0.015	0.784 ± 0.024	N/A
Golilarz 2023	CNN-KCL	4686	0.746 ± 0.031	0.836 ± 0.024	0.733 ± 0.034	0.802 ± 0.025	0.739 ± 0.031	0.79 ± 0.026	N/A
Golilarz 2023	RLMD-PA	4686	0.844 ± 0.014	0.893 ± 0.024	0.827 ± 0.033	0.874 ± 0.016	0.835 ± 0.019	0.868 ± 0.014	N/A
Golilarz 2023	MD	4686	0.81 ± 0.017	0.852 ± 0.009	0.792 ± 0.018	0.841 ± 0.014	0.804 ± 0.007	0.84 ± 0.012	N/A
Golilarz 2023	GAN-MD	4686	0.898 ± 0.018	0.931 ± 0.032	0.883 ± 0.051	0.912 ± 0.03	0.862 ± 0.032	0.91 ± 0.035	N/A
Golilarz 2023	AlexNet	4686	0.709	0.832	0.718	0.786	0.713	0.768	N/A
Golilarz 2023	GoogleNet	4686	0.794	0.765	0.671	0.776	0.727	0.779	N/A
Golilarz 2023	ResNet	4686	0.727	0.784	0.67	0.763	0.697	0.755	N/A
Golilarz 2023	DenseNet	4686	0.741	0.758	0.648	0.751	0.691	0.749	N/A
Golilarz 2023	MobileNet	4686	0.754	0.801	0.696	0.783	0.724	0.777	N/A
Golilarz 2023	WCE	4686	0.759	0.864	0.771	0.825	0.765	0.81	N/A
Golilarz 2023	BCE	4686	0.821	0.814	0.734	0.817	0.775	0.817	N/A
Golilarz 2023	DL	4686	0.807	0.801	0.71	0.803	0.755	0.804	N/A
Golilarz 2023	TL	4686	0.794	0.822	0.728	0.811	0.76	0.808	N/A
Golilarz 2023	GAN	4686	0.782	0.825	0.729	0.809	0.755	0.803	N/A
Golilarz 2023	DRAGAN	4686	0.842	0.887	0.818	0.87	0.83	0.864	N/A
Golilarz 2023	AGE	4686	0.715	0.758	0.64	0.742	0.675	0.736	N/A
Golilarz 2023	α-GAN	4686	0.814	0.845	0.76	0.833	0.786	0.829	N/A
Danaei 2022	CNN-KCL	586	0.752 ± 0.026	0.842 ± 0.021	0.742 ± 0.03	0.808 ± 0.021	0.747 ± 0.026	0.796 ± 0.021	N/A
Danaei 2022	RLMD-PA	586	0.852 ± 0.018	0.897 ± 0.025	0.834 ± 0.034	0.88 ± 0.018	0.842 ± 0.021	0.874 ± 0.016	N/A
Danaei 2022	CNN + BA + RL	586	0.814 ± 0.047	0.88 ± 0.009	0.803 ± 0.014	0.855 ± 0.017	0.808 ± 0.027	0.846 ± 0.023	N/A
Danaei 2022	CNN + DE + RL	586	0.831 ± 0.03	0.882 ± 0.03	0.81 ± 0.04	0.863 ± 0.024	0.82 ± 0.03	0.856 ± 0.024	N/A
Danaei 2022	CNN + ABC + RL	586	0.871 ± 0.022	0.91 ± 0.025	0.854 ± 0.036	0.895 ± 0.018	0.862 ± 0.022	0.89 ± 0.016	N/A
Danaei 2022	WCE	586	0.762	0.871	0.78	0.83	0.771	0.815	N/A
Danaei 2022	BCE	586	0.822	0.818	0.739	0.819	0.778	0.82	N/A
Danaei 2022	FL	586	0.837	0.876	0.803	0.861	0.82	0.856	N/A
Danaei 2022	DE	586	0.816	0.803	0.713	0.807	0.761	0.809	N/A
Danaei 2022	TL	586	0.801	0.823	0.731	0.815	0.764	0.812	N/A
Noto 2019	KNN (2D)	62	0.91 ± 1	0.8 ± 0.03	0.89 ± 0.01	0.87 ± 0.01	N/A	N/A	N/A
Noto 2019	LDA (2D)	62	0.93 ± 0.01	0.8 ± 0.03	0.89 ± 0.01	0.88 ± 0.01	N/A	N/A	N/A
Noto 2019	Multilayer perceptron NN (2D)	62	0.9 ± 0.01	0.84 ± 0.03	0.91 ± 0.01	0.88 ± 0.01	N/A	N/A	N/A
Noto 2019	SVM (2D)	62	0.93 ± 0.01	0.82 ± 0.04	0.9 ± 0.02	0.89 ± 0.01	N/A	N/A	N/A
Noto 2019	TB (RF) (2D)	62	0.9 ± 0.01	0.84 ± 0.02	0.91 ± 0.01	0.87 ± 0.01	N/A	N/A	N/A
Noto 2019	KNN (3D)	62	0.94 ± 0.02	0.61 ± 0.03	0.81 ± 0.01	0.82 ± 0.01	N/A	N/A	N/A
Noto 2019	LDA (3D)	62	0.94 ± 0.01	0.8 ± 0.02	0.89 ± 0.01	0.89 ± 0.01	N/A	N/A	N/A
Noto 2019	Multilayer perceptron NN (3D)	62	0.9 ± 0.01	0.8 ± 0.02	0.89 ± 0.01	0.87 ± 0.01	N/A	N/A	N/A
Noto 2019	SVM (3D)	62	0.89 ± 0.02	0.74 ± 0.02	0.86 ± 0.01	0.83 ± 0.01	N/A	N/A	N/A
Noto 2019	TB (RF) (3D)	62	0.85 ± 0.02	0.68 ± 0.04	0.83 ± 0.02	0.79 ± 0.02	N/A	N/A	N/A
Kaya 2024	GPT-4	171	0.9	0.78	0.76	0.83	0.82	N/A	N/A
Wang 2024a	Three ML - Internal	87	0.966 (95% CI 0.923-1.000)	0.971 (95% CI 0.922–0.993)	N/A	N/A	0.724 (95% CI 0.638–0.795)	N/A	0.987 (95% CI 0.978–0.995)
Wang 2024b	Three ML - External	66	0.955 (95% CI 0.855-1.000)	0.955 (95% CI 0.885-1.000)	N/A	N/A	0.630 (95% CI 0.514–0.735)	N/A	0.972 (95% CI 0.95–0.989)

Regarding validation method, external validation of the ML models was used only in one study, raising potential bias concerns in remaining studies.

Most of the included papers (8) assessed diagnostic performance of ML models on Iranian-based Z-Alizadeh Sani myocarditis dataset. Reference standard (gold standard) standard usually involved physicians experienced with CMR imaging, however this information was not available in Z-Alizadeh Sani myocarditis dataset [21, 22]. Notably, this dataset included only myocarditis and healthy images. The study from Wang and al. assessed ML diagnosis on 12 different conditions, surpassing any other study in terms of number of conditions [26]. The study from Zaman et al. had 5 different conditions in the dataset, while other papers had only 2 conditions, including myocarditis [18].

Table 2 summarizes diagnostic accuracy of 141 models included in this systematic review. Mean sensitivity was 0.819 ± 0.089, specificity 0.837 ± 0.114, precision 0.773 ± 0.128, accuracy 0.830 ± 0.093, F-score 0.782 ± 0.108, G-means 0.797 ± 0.075, and mean AUC value was 0.923 ± 0.110.

### Diagnostic accuracy Meta-Analysis of all models

**Fig. 2:**
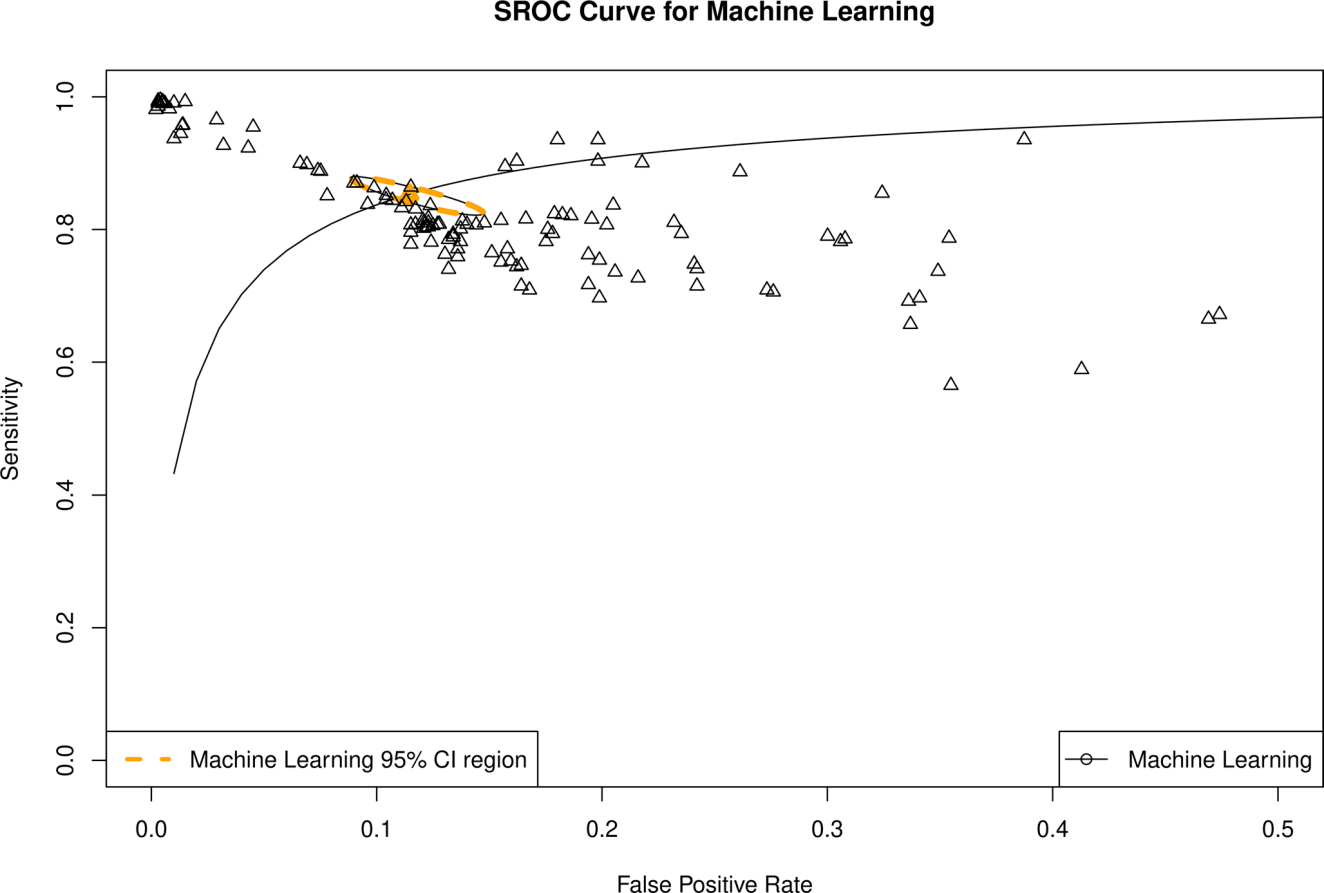
Summary ROC of overall DTA meta-analysis

**Fig. 3:**
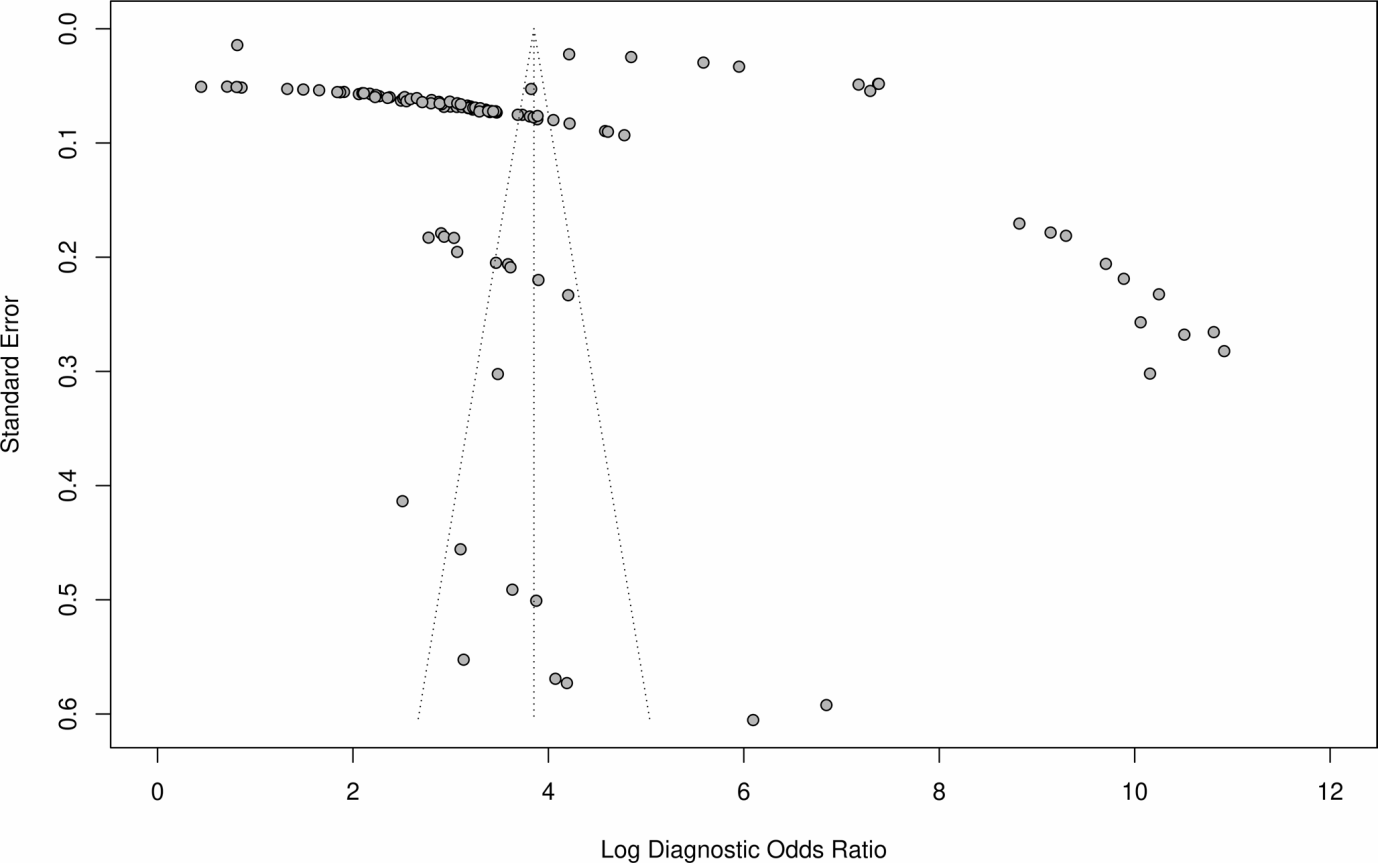
Funnel plot for overall DTA meta-analysis

Bivariate analysis yielded an AUC of 0.93 (95% CI 0.90–0.94; Fig. 2), sensitivity of 0.85 (95% CI 0.83–0.881), false positive rate of 0.12 (95% CI 0.09–0.14), and DOR 47.1 (95% CI 31.3–70.9). Threshold analysis was present − 86.9%. High I^2^ value (99.9%, *p* < 0.005) was observed. Publication bias was observed by funnel plot assymetry and confirmed through Deek’s test (*p* < 0.001; Fig. 3).

In the univariate analysis, pooled sensitivity was 0.86 (95% CI 0.84–0.88; I^2^ = 99.8%, *p* < 0.005), while specificity 0.88 (95% CI 0.86–0.91; I^2^ = 99.8%, *p* < 0.005),

### Diagnostic accuracy Meta-Analysis of best performing models

**Fig. 4:**
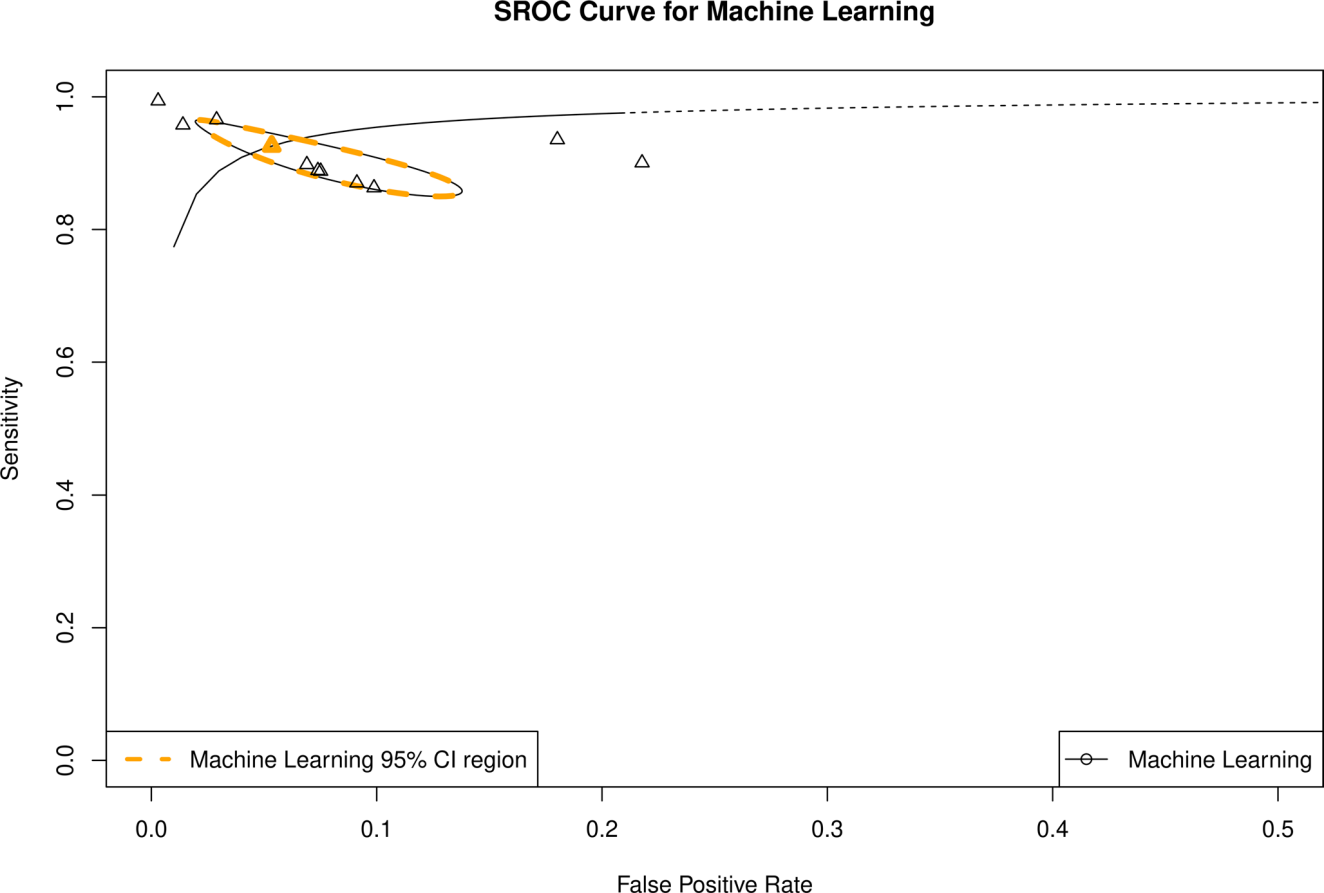
Summary ROC for best-performing models

**Fig. 5:**
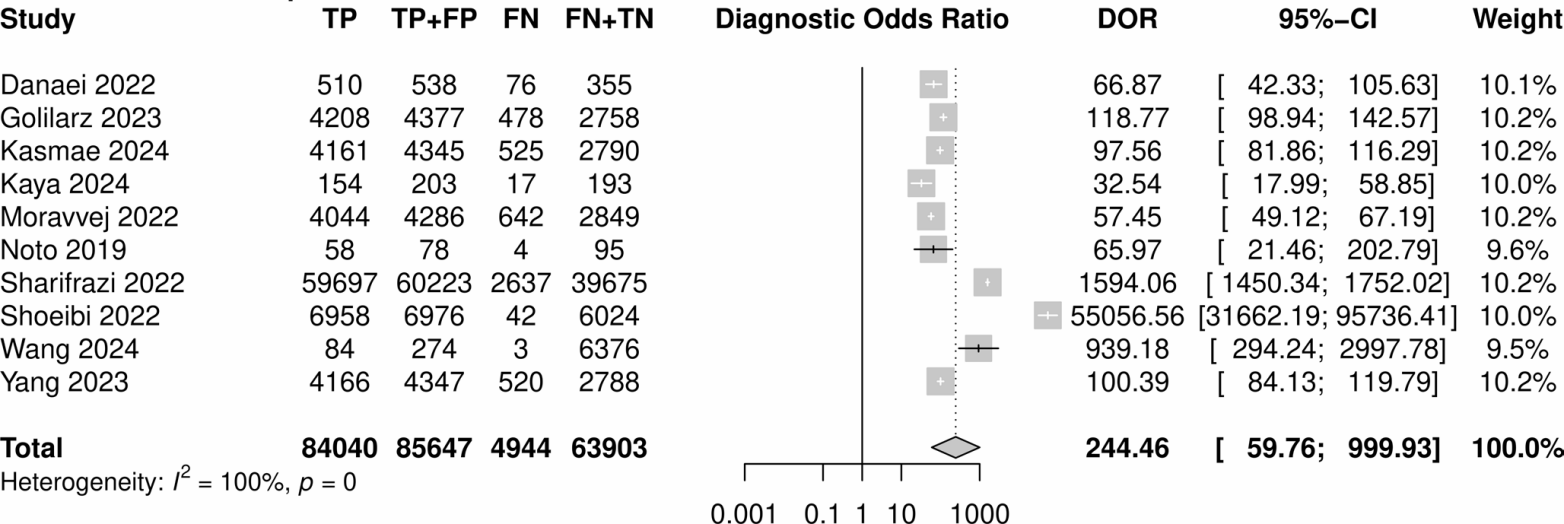
DOR forest plot for best-performing models

DTA for the best model from each study showed AUC of 0.97 (95% CI 0.93–0.98; Fig. 4), sensitivity of 0.93 (95% CI 0.87–0.96), false positive rate of 0.05 (95% CI 0.02–0.12), and DOR of 244.5 (95% CI 59.8-999.3; Fig. 5). The presence of the threshold effect was again confirmed. High heterogeneity was observed (I^2^ = 99.6%, *p* < 0.005).

**Fig. 6:**
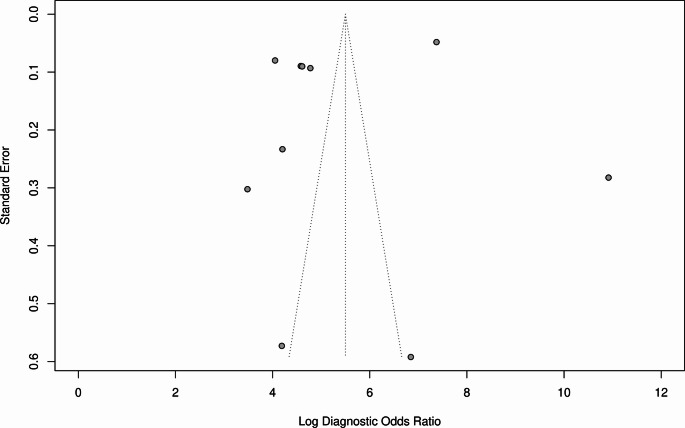
Funnel plot for best-performing models

Some asymmetry was observed with funnel plot analysis. However, Deek’s funnel plot asymmetry test did not confirm the presence of publication bias (*p* = 0.26; Fig. 6).

**Fig. 7:**
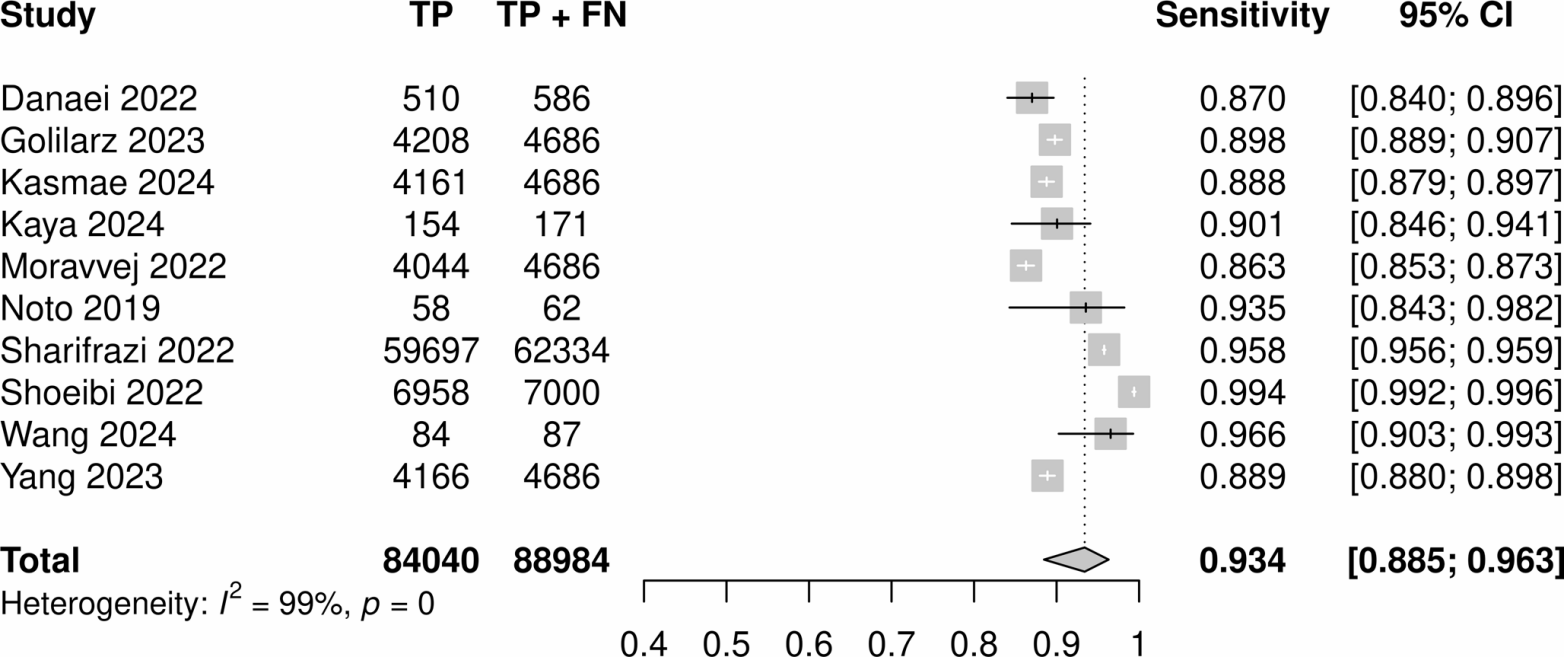
Univariate sensitivity forest plot for best performing models from each study

**Fig. 8:**
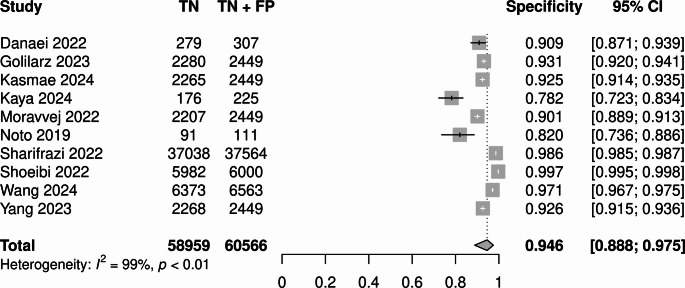
Univariate specificity forest plot for best performing models from each study

Univariate analysis showed sensitivity of 0.93 (95% CI 0.88–0.96; I^2^ = 99.4%, *p* < 0.005; Fig. 7), and specificity of 0.95 (95% CI 0.89–0.97; I^2^ = 99.3%, *p* < 0.01; Fig. 8).

### Evaluation of ML models to human physicians and comparison of diagnostic accuracy

The summary of comparisons is described in Table 3. Noto et al. have compared ML classifiers for myocardial infarction (MI) and myocarditis classification to one reader with one year with CMR experience and a second reader with five years with CMR experience. A testing set based on 10-fold cross validation on 173 patients was performed. The best performing model on 2D analysis (SVM) achieved 93% of sensitivity, 84% of specificity, and 89% of accuracy. The less experienced reader achieved 63%, 42%, and 55%, while the experienced reader achieved 95%, 96%, and 95% of sensitivity, specificity, and accuracy, respectively.

**Table 3:** Comparison between the best ML model and clinicians

Study	Aim	Machine Learning					Expert					p-value
		Model	Sensitivity	Specificity	Accuracy	F-Score	Expert	Sensitivity	Specificity	Accuracy	F-Score	
Noto 2019	Myocarditis and MI differentiation (2D analysis)	SVM (2D)	0.93	0.84	0.89	N/A	One year of CMR experience	0.63	0.42	0.55	N/A	N/A
							Five years of CMR experience	0.95	0.96	0.95	N/A	N/A
Noto 2019	Myocarditis and MI differentiation (3D analysis)	LDA (3D)	0.94	0.8	0.89	N/A	One year of CMR experience	0.66	0.76	0.69	N/A	N/A
							Five years of CMR experience	0.97	0.95	0.96	N/A	N/A
Kaya 2024	Myocarditis and no Myocarditis differentiation	GPT-4	0.9	0.78	0.86	0.82	One year of CMR experience	0.9	0.84	0.86	0.85	*p* = 0.14
							Two years of CMR experience	0.86	0.91	0.89	0.87	*p* = 0.007
							Four years of CMR experience	0.85	0.96	0.91	0.89	*p* < 0.001
Wang 2024	Classification of eleven CVDs	CNN based model with leaky rectified linear unit (ReLU)	N/A	N/A	N/A	0.857	Three to five years of CMR experience	N/A	N/A	N/A	0.553	N/A, authors state that in all cases, ML was superior
							Five to ten years of CMR experience				0.6	
							More than ten years of CMR experience				0.683	

In the 3D analysis of CMR, the best performing ML model (LDA) scored 94% of sensitivity, 80% of specificity, and 89% of accuracy. First reader achieved 66%, 76% and 69%, while expert reader achieved 97%, 95%, and 96%.

Kaya et al. have compared the effectiveness of CMR reports from eight tertiary care medical centers in Germany between GPT-4 chat and three radiology residents with various experience in cardiovascular imaging. These reports included radiology, demographic, and clinical data, and were further compared with a reference (gold) standard with cardiovascular imaging experts. The task was to distinguish between Myocarditis and non-Myocarditis cases.

On a dataset with 396 cases, GPT-4 ML chat achieved sensitivity of 90%, specificity of 78%, and accuracy of 83%. This was comparable with a resident with just one year of experience (90%, 84%, and 86%, *p* = 0.14), however inferior to results achieved by a resident with two years of experience (86%, 91%, and 89%, *p* = 0.007) and four years of experience (85%, 96%, and 91%, *p* < 0.001).

Finally, Wang and colleagues analyzed the performance of their CNN model and compared the effectiveness of diagnosis of eleven cardiovascular diseases (CVDs) between ML model and physicians with various CMR imaging experience. They developed a separate testing set with 500 patients. Physicians in each group worked independently to classify CVDs. CNN achieved a higher F-score in diagnosis of myocarditis (0.857), when compared to physicians with three to five years of CMR experience (0.553), five to ten years of CMR experience (0.600), and more than ten years of CMR experience (0.683). Notably, the algorithm was several times faster in completing the classification task, when compared to human experts (1.94 min vs. 418 min for classification of 500 patients).

### Quality assessment (Risk of Bias)

**Fig. 9:**
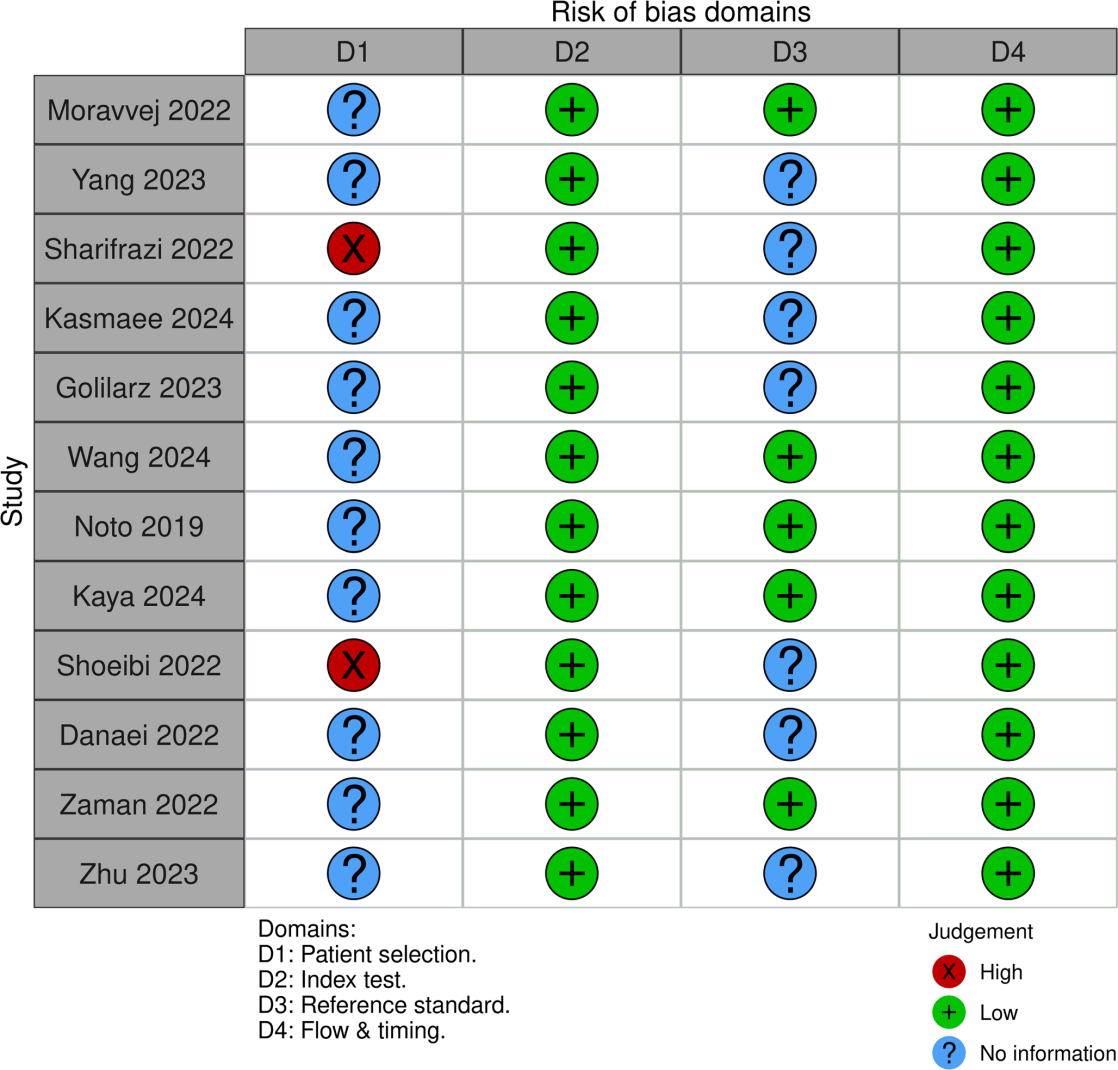
Quality assessment of the included studies. Risk of bias was assessed in four domains with QUADAS-2 tool

A quality assessment of the included studies was performed independently (blinded) by two authors with QUADAS-2 tool. Overall, the quality assessment revealed a moderate level of heterogeneity in the quality of the included studies. Figure 9 presents a traffic light plot that visually summarizes the risk of bias across all domains for each study.

## Discussion

### The summary of comparison between ML-Based diagnosis and human physicians

This systematic review DTA meta-analysis demonstrate that ML models, particularly custom-tuned CNNs with various layers, focal, loss function, and cluster modifications, can achieve high diagnostic accuracy, even exceeding the performance of human cardiac imaging experts, especially those with less experience in CMR imaging. However, the main advantages do not end there, as ML models offer also additional benefits, including significant improvements in processing time of CMR datasets, and analyzing large datasets significantly faster than human readers. This potentially allows diagnosing patients much faster in overloaded medical centers. Additionally, rapid diagnosis may be used with many patients who are suspected of having acute myocarditis, and need rapid clinical decision-making for next treatment steps. ML models could be especially useful, due to their rapidity.

### ML versus human performance

Several studies have evaluated head-to-head diagnostic accuracy of ML models to human readers. Wang et al.‘s study highlights the potential of CNNs to outperform physicians across varying levels of CMR experience in diagnosing myocarditis and other CVDs [26]. The reported F1-score of 0.857 for CNNs compared to 0.553–0.683 for physicians underscores this potential. The significant reduction in processing time (1.94 min vs. 418 min for 500 cases) can be especially beneficial in high-volume medical centers in major cities.

Interestingly, the comparison of GPT-4’s performance with radiology residents by Kaya et al. provides a valuable benchmark against human interpretation [28]. While GPT-4 matched the performance of a 1-year resident, its underperformance compared to more experienced residents suggests that further development is needed to achieve expert-level accuracy. LLMs are constantly evolving, and compared to CNNs or other traditional ML algorithms, do not require programming knowledge to use them for diagnosis. These web-based, highly accessible, and relatively popular algorithms are becoming more popular in recent years, and could enhance text-based diagnosis from CMR reports, or even image diagnosis, when image analysis function will be implemented within LLMs.

Noto et al.‘s findings reinforce the potential of ML models to achieve high accuracy in both 2D and 3D CMR image analysis [27]. The superior performance of the SVM and LDA models compared to less experienced human readers suggests that ML could play a crucial role in supporting clinical decision-making, particularly in settings with limited access to expert CMR interpretation. As mentioned earlier, the ML-aided diagnosis could also improve novice resident accuracy, minimizing potential false positive or false negative results. The reduction of false diagnosis could be potentially lifesaving with patients who have acute subtypes, and also save potential hospitalization costs in case of misdiagnosis of myocarditis. The minimization of false diagnosis could also improve a patient’s mental condition.

### The summary of diagnostic accuracy

A total of 122 models were included in the diagnostic accuracy meta-analysis, which yielded sensitivity of 0.86 (95% CI 0.84–0.88) and specificity of 0.88 (95% CI 0.86–0.91), from ML-based CMR diagnostic studies. Such diagnostic values support the high accuracy of ML models for myocarditis diagnosis. Additionally, high summary ROC AUC value was observed from bivariate analysis − 0.93 (95% CI 0.90–0.94). On the other hand, while the DTA results are promising, relatively high heterogeneity was observed among performed analyses (I² >95%), which requires further testing, before the AI algorithms become widely applied in a clinical scenario. While threshold effect was observed in the meta-analysis, variations in study design, size of images and length of CMR reports, various patient populations, prevalence of myocarditis cases, inclusion of various cardiovascular diseases in the training and validation sets, different CMR protocols, validation techniques, experience of the labelling staff, and ML model architectures likely contribute as well to between-study variations. Additionally, publication bias was present in the overall analysis (*p* < 0.001).

For the best 10 models, one from each eligible study, pooled sensitivity and specificity were even higher, 0.93 (95% CI 0.88–0.96) and 0.95 (95% CI 0.89–0.97), respectively. However, high heterogeneity was again observed in the analyses, due to aforementioned reasons.

### Limitations and future steps for ML-CMR implementation for myocarditis diagnosis

Assistance of ML in diagnosis holds an enormous clinical potential, aiding residents, and inexperienced radiologists. However, there are several crucial limitations observed in the included trials from this meta-analysis.

### Heterogeneity

One of the major limitations is the very high heterogeneity of the results. This was caused, as the included studies varied significantly in terms of the specific ML algorithms designs, size of CMR images, heterogeneous patient populations, different prevalence of myocarditis cases in the datasets for training and validation, inclusion of various non-myocarditis conditions for the diagnostic accuracy analysis in the training and validation sets, varying imaging protocols and validation techniques, and different data augmentation techniques. Such variability had highly influenced the overall pooled estimates of diagnostic accuracy.

To reduce the heterogeneity of the results, future studies need a commonly adopted standardization for full-scale the implementation of ML-CMR techniques [29]. This process will potentially require transparent guidelines for cardiac imaging datasets developers. Additionally, guidelines for the ML engineers are needed, to capture most relevant imaging or report parameters to develop high diagnostic accuracy models, which could be applied in different healthcare facilities.

### Lack of gold standard transparency

Another aspect is the proper gold standard. Studies should transparently report who performed labelling of the myocarditis patient - the non-perfect gold standard may lead to misleading results and wrongly trained ML model. In some studies, details who performed final labelling was unclear. This process should ideally be performed by multiple CMR experts independently, to minimize potential bias resulting from subjective opinion. Assistance of arbiters in case of disagreements should also be considered in such situation.

### Inclusion of demographic factors

Additionally, we observed in most of the studies a lack of demographic summaries of the dataset (patients), which could enhance the model development itself, by adding relevant clinical parameters. Different baseline demographic information could influence the performance of the model for specific subpopulations. ML models could apply demographic details to improve final diagnosis of the myocarditis. Inclusion of previous cardiovascular incidents, history of treatment, and genetic risk could enhance accuracy of algorithms.

### Variations and lack of transparency in CMR protocols

CMR imaging protocols, which were used to develop the imaging dataset, also eed to be provided, not only for dataset owners, but also for the future authors, who might use (in full or partially with other datasets) imaging databases to develop novel mode The increasing number of myocarditis databases allows for even more robust diagnostic accuracy, as ML models cnls.an apply training data from online sources, and constantly improve diagnostic performance over time. For the software engineers, detailed information about CNNs parameters, mathematical functions used in the algorithm, or application of advanced approaches could be useful in improvement of current models [30]. Another aspect is the preparation of the data itself - studies varied how images were prepared, but common techniques included color normalization, resizing and rotation of the images, or noise reduction. The potential standardization of data would make the datasets less heterogeneous, therefore making overall heterogeneity between studies lower due to the comparable data augmentation techniques [31]. This could also provide more reliable comparisons and pave the future way to determine which ML models are the best, after head-to-head comparisons.

### Limitations in datasets

Another significant limitation is that the majority of the included studies are single-center/single-dataset based, which raises concerns about the real clinical performance of the algorithms. The reliance on small number of patients from a single center could lead to overoptimistic results of the model. Larger, multicenter studies with diverse patient populations, various subtypes of myocarditis condition, and clinical settings are needed to validate the performance of ML-CMR in diagnosing myocarditis across different healthcare environments. Wang et al. have performed validation of their model not only on the dataset from multiple healthcare institution, but also externally validated their model on independent, unseen dataset [26].

### Lack of application in clinical practice with simple, two-codition diagnosis

Also, most of the studies had not specified the subtype of myocarditis, which is crucial for clinical implementation. Studies in this meta-analysis focused on simple diagnosis between myocarditis and non-myocarditis images. This approach is again highly overoptimistic, and does not reflect clinical conditions, where patients are admitted with various cardiovascular problems in the clinic. The proportion of the myocarditis cases in the datasets and testings sets was also adjusted for model training and development. The prevalence of myocarditis in clinical scenario is lower, therefore researchers should be able to recognize not only the fact that incidence of this specific disease is typically lower than in experimental studies presented in this meta-analysis, but there are many more diverse conditions with various incidence rates. Therefore, multi-cardiovascular disease models should be implemented to reflect the typical clinical settings, as two-condition only algorithms are not suited for diagnostic applications in healthcare facilities.

### Overreliance on single dataset

Another observed concern is the reliance on single dataset, in this review the Z-Alizadeh Sani dataset. This dataset of myocarditis images was used in multiple studies, and raises concerns about generalizability. It contains only two classes in the training and validation sets—myocarditis and healthy controls—which creates an artificially simplified, binary classification problem. Such approach does not reflect the real clinical scenario, where myocarditis must be distinguished from other pathologies like myocardial infarction or other cardiomyopathies, and non-cardiology pathologies. Additionally, these datasets did not analyze subtypes of myocarditis, such as acute, chronic, or fulminant. Such simplicity could lead to artificially high DTA values, which may not align with clinical scenarios.

Studies like Wang et al.‘s, which incorporate a broader range of cardiovascular conditions and imaging protocols, offer a more robust evaluation of ML model performance, reflecting a typical clinical scenario.

### Lack of transparency in software/hardware used

Furthermore, authors of the included studies did not provide detailed reporting on hardware and software specifications used for model development. This crucial aspect hinders reproducibility and comparability across studies. Authors of the future studies should take this into consideration, as future developers may compare the most optimal approaches between the studies. This issue could be resolved by implementation of the guidelines in ML for cardiovascular imaging.

### Black box concern

The black box issue is a common problem for ML algorithms applied in healthcare [32]. ML algorithms may perform clinical decisions, without clear information on how this decision (diagnosis) was processed. This becomes especially problematic in a case, when clinicians need to base final decision with ML assistance. Neither patient, software engineer, nor physician understand how this input/output process influenced the final decision. This issue is currently underestimated and should be given priority for future ML algorithms development [32, 33].

### Limited human comparison

While we provided a simple evaluation between ML algorithms and human physicians, due to insufficient data available from the studies, more comprehensive comparison was not possible. The aim of future studies should be to report more comprehensive evaluations to humans, especially between different specialty/experience levels. Another issue is about cost-effectiveness, this was not performed, due to insufficient data from the studies. Such analysis could be impactful, especially for policymakers, as their priority is to introduce cost-effective technology into healthcare centers.

### Limited external validation

Finally, external validation is essential to ensure that ML models perform reliably in real-world clinical settings. The limited use of external validation in the included studies underscores the need for greater emphasis on this aspect of model development. Internal validation could provide too optimistic results of the ML algorithms [34].

The promising diagnostic performance demonstrated by CNNs and the benchmarking of GPT-4 against radiology residents pave the way for targeted enhancements in ML applications [26, 28]. However, the gap between research and clinical practice needs to be filled with data from future studies, that should focus on real-time prospective deployment and user-centered design. While high diagnostic accuracy is essential for myocarditis diagnosis, clinicians require systems that can be easily applied into existing workflows, provide interpretable results with clear explanation how decision (diagnosis) was performed, work in different hospital conditions (i.e., imaging protocols and healthcare IT systems), and adapt to varying resource constraints across healthcare settings.

Transparency could be enhanced by the development of explainable artificial intelligence (XAI) approaches, such as saliency maps or case-based reasoning tools [35]. For example, ML algorithms could visually highlight the specific myocardial regions that most influence diagnostic predictions, helping clinicians correlate ML outputs with pathophysiological insights [36–40]. This could be a solution for the existing black box issue, which is currently a major problem in terms of liability of the physician.

Imbalance in the categories (the number of myocarditis images) can influence the performance of CNNs [17, 41]. ML developers should be aware of this issue, as in clinical scenarios there is disproportion between patients with normal, abnormal, and myocarditis cases. Inclusion of various cardiovascular conditions is essential for rigorous model testing and for real-life clinical application to prevent potential misdiagnoses of the patients.

Another promising avenue is to expand the role of ML beyond diagnosis to guide therapy selection [42, 43]. Models could incorporate biomarkers, clinical history, and imaging data to stratify patients by risk or predict responses to treatments like immunosuppressive therapies. These predictive capabilities could revolutionize personalized treatment strategies for myocarditis, improving patient outcomes.

Ethical concerns remain paramount, particularly regarding data security, potential biases, and the transparency of ML algorithms [44–47]. Although included studies provided details on demographic information, these studies failed to inform whether racial or gender biases were encountered during model training or testing. It is important to know that ML models are highly dependent on the training data. Homogenous populations may lead to poor results in clinical practice, as patients with diverse demographics might be encountered. Therefore, comprehensive, multi-center datasets are needed to minimize the impact of bias in the validation.

Institutional review boards and regulatory agencies should establish clear frameworks for deploying ML models in clinical settings. Concurrently, operational hurdles such as interoperability with existing imaging platforms and compatibility with electronic health record systems must be prioritized to facilitate smooth adoption.

## Conclusion

While the current meta-analysis suggests that some ML-powered CMR techniques demonstrate high diagnostic accuracy for myocarditis, further research is warranted to address the identified limitations and expand the clinical evidence supporting the integration of these advanced imaging approaches into routine clinical practice.

## Data Availability

No datasets were generated or analysed during the current study.
